# Immunonutritional Indices, Inflammatory Markers, and Thyroid-Related Parameters in Adults with Hashimoto’s Thyroiditis

**DOI:** 10.3390/nu18111698

**Published:** 2026-05-26

**Authors:** Hulya Yilmaz Onal, Songul Aktas, Aysun Yuksel, Tutku Tuncalı Yaman, Ozcan Keskin, Hafize Uzun

**Affiliations:** 1 Department of Nutrition and Dietetics, Faculty of Health Sciences, Istanbul Medeniyet University, Istanbul 34862, Turkey; hulya.onal@medeniyet.edu.tr (H.Y.O.); aysun.yuksel@medeniyet.edu.tr (A.Y.); 2 Department of Internal Medicine, Kartal Dr. Lutfi Kirdar City Hospital, Istanbul 34865, Turkey; drsongulbahadir@gmail.com (S.A.); okeskin34@yahoo.com (O.K.); 3 Knappschafts-Klinik Bad Driburg, 33014 Bad Driburg, Germany; 4 Department of Management Information Systems, Faculty of Business Administration, Marmara University, Istanbul 34854, Turkey; tutku.tuncali@marmara.edu.tr; 5 Department of Medical Biochemistry, Faculty of Medicine, Istanbul Atlas University, Istanbul 34403, Turkey

**Keywords:** Hashimoto’s thyroiditis, immunonutritional indices, PNI, CONUT, NRI, systemic inflammation, vitamin D

## Abstract

**Background:** Hashimoto’s thyroiditis (HT) is a chronic autoimmune disorder characterized not only by thyroid dysfunction but also by metabolic disturbances, micronutrient inadequacies, and low-grade inflammation. Composite indices derived from routine laboratory parameters may therefore help capture the broader systemic profile of the disease. This study explored within-cohort associations of immunonutritional indices including the Prognostic Nutritional Index (PNI), Nutritional Risk Index (NRI), and Controlling Nutritional Status (CONUT), and hemogram-derived inflammatory markers including the Neutrophil-to-Lymphocyte Ratio (NLR), Monocyte-to-Lymphocyte Ratio (MLR), Platelet-to-Lymphocyte Ratio (PLR), and Systemic Immune-Inflammation Index (SII), with thyroid function, thyroid autoimmunity, metabolic characteristics, disease duration, and vitamin D status in adults with Hashimoto’s thyroiditis. **Methods:** This cross-sectional study included 229 adults diagnosed with HT. PNI, NRI, CONUT, and complete blood count-derived inflammatory markers were evaluated in relation to thyroid function, thyroid autoimmunity, disease duration, metabolic characteristics, and vitamin D status. Because most variables were not normally distributed, the main analyses were conducted using non-parametric tests. Correlations were evaluated using Spearman’s rank correlation coefficients. Exploratory regression models were estimated using HC3 heteroscedasticity-consistent robust standard errors, and CRP-based sensitivity analyses were performed by excluding participants with CRP > 10 mg/L. **Results:** Vitamin D deficiency was highly prevalent and affected 70.3% of the participants. Among the immunonutritional indices, NRI differed significantly according to BMI category and HOMA-defined insulin resistance (both *p* < 0.001), indicating a closer relationship with metabolic burden. PNI was associated with disease duration (*p* = 0.009), whereas the inflammatory indices were largely similar across the clinical groupings examined. In exploratory robust regression models, the explanatory power remained modest (R^2^ = 0.066–0.171). PLR showed the most consistent index-related association with TSH, whereas the CONUT–FT3 association observed in the full-sample robust model was not retained after CRP-based sensitivity analysis. **Conclusions:** Adults with HT in this study showed frequent vitamin D deficiency together with a substantial burden of excess weight and insulin resistance. Routine immunonutritional and inflammatory indices may provide supportive information on within-cohort biochemical and metabolic heterogeneity, but they should not be interpreted as stand-alone diagnostic or prognostic markers. In particular, NRI appeared to reflect metabolic and adiposity-related burden more than nutritional risk alone, while PLR showed the most internally consistent index-related association with TSH.

## 1. Introduction

Hashimoto’s thyroiditis (HT) is a chronic, organ-specific autoimmune inflammatory disease and one of the most common causes of hypothyroidism [1,2,3]. Community-based epidemiological data indicate that autoimmune thyroid disease constitutes a substantial disease burden and has a higher prevalence in women than in men [4]. In HT, anti-thyroid peroxidase (anti-TPO) and anti-thyroglobulin (anti-TG) antibodies target thyroid tissue, thereby triggering the autoimmune response and inflammation. Lymphocytic infiltration is characteristic on histopathology, and ultrasonographic findings suggestive of this condition are frequently observed. Although thyroid-stimulating hormone (TSH) and free thyroxine (FT4) remain central to the diagnostic approach, they may be limited in comprehensively reflecting the extra-thyroidal systemic manifestations of Hashimoto’s thyroiditis, particularly inflammatory activity and metabolic burden [1,2].

Complete blood count-derived inflammatory markers (Neutrophil-to-Lymphocyte Ratio (NLR), Monocyte-to-Lymphocyte Ratio (MLR), Platelet-to-Lymphocyte Ratio (PLR), and Systemic Immune-Inflammation Index (SII)) are increasingly used as accessible indicators of inflammatory activity in HT [5]. Analyses based on the National Health and Nutrition Examination Survey (NHANES) have reported significant associations between SII and thyroid function indicators in U.S. adults; these findings suggest that systemic inflammatory status may be linked to thyroid hormone profiles [6]. Similarly, population-based findings reporting an association between SII and thyroid function also point to a possible link between systemic inflammation and thyroid hormone profiles [7]. Moreover, recent clinical studies in HT have reported higher NLR/PLR and related indices compared with control groups; these results suggest that such indices may be clinically useful as low-cost and readily available inflammatory markers [5,8]. Autoimmune thyroid disease is also associated with impairments in metabolic and nutritional status; therefore, nutritional factors are increasingly considered a component of the clinical and biological framework of the disease [9,10,11]. Accordingly, assessing immunonutritional status in addition to inflammatory indices may provide a more comprehensive perspective on the systemic features of HT. However, these indices are not specific to autoimmune thyroid disease and should be interpreted as accessible supportive markers of systemic inflammatory status rather than disease-specific diagnostic indicators.

Beyond classical thyroid autoantibodies, HT is characterized by an interplay in which immune activation and tissue-specific damage mutually influence each other and may exhibit marked variability across patients [1,12]. The clinical presentation can range from asymptomatic euthyroidism to overt hypothyroidism, suggesting a broad phenotypic spectrum in HT [1,2]. Accordingly, heterogeneity in thyroid function tests within clinically diagnosed HT populations should be interpreted as reflecting this spectrum rather than as evidence against the diagnosis itself. Given this heterogeneity, evaluating immunonutritional status alongside complete blood count-derived inflammatory ratios may contribute to a more comprehensive characterization of clinical and biochemical variability in HT [1,12].

Metabolic burden is a prominent feature of autoimmune thyroid disease and is closely related to nutritional status [9,11]. In HT cohorts, various nutrient deficiencies have been reported, including insufficiencies in vitamins and trace elements that are critical for thyroid metabolism and immune regulation [10,11]. These findings support the nutrition–immune axis approach and indicate that undernutrition or poor diet quality may contribute to immune dysregulation in HT by increasing oxidative stress [9].

To render this approach clinically measurable, immunonutritional indices such as the Prognostic Nutritional Index (PNI), Nutritional Risk Index (NRI), and Controlling Nutritional Status (CONUT) score provide a summary indicator of nutritional and immune status by combining components such as albumin, lymphocyte count, and body weight [13,14,15]. Although these indices are widely used in different clinical contexts, their use in HT is limited, and their relationships with thyroid autoimmunity and metabolic characteristics have not been clearly established [1]. This knowledge gap is important because practical and low-cost indices may complement standard thyroid hormone tests and help identify patients with a higher inflammatory burden or nutritional insufficiency [9]. The number of studies jointly evaluating inflammatory and immunonutritional indices in HT remains limited, creating uncertainty regarding how these measures relate to thyroid function indicators, autoimmunity markers, and associated metabolic features in routine practice [1,5]. Therefore, systematic evaluation of these composite markers together with standard thyroid tests appears clinically meaningful [1,2].

Studies that address complete blood count-derived inflammatory ratios together with immunonutritional indices in HT remain limited in the current literature. Therefore, the aim of this study was to explore within-cohort associations between immunonutritional indices (PNI, NRI, and CONUT), hemogram-derived inflammatory markers (NLR, MLR, PLR, and SII), thyroid function, thyroid autoimmunity, metabolic characteristics, disease duration, and vitamin D status in adults with clinically diagnosed Hashimoto’s thyroiditis.

## 2. Materials and Methods

### 2.1. Study Design and Population

This cross-sectional study included 229 volunteer patients who attended the internal medicine outpatient clinic of Istanbul Kartal Dr. Lütfi Kırdar City Hospital between May 2023 and December 2023. A structured questionnaire was administered to all participants, and demographic characteristics, together with anthropometric measurements, including body weight and height, were recorded.

### 2.2. Inclusion Criteria and Exclusion Criteria

Patients aged 18–65 years with a confirmed diagnosis of HT, supported by laboratory findings including anti-thyroid peroxidase (anti-TPO), anti-thyroglobulin (anti-TG), and thyroid ultrasonography, were eligible for inclusion. Exclusion criteria were pregnancy, lactation, active infection, malignancy, chronic renal failure, hemodialysis or peritoneal dialysis treatment, advanced heart failure (New York Heart Association class III–IV), and chronic lung disease, including chronic obstructive pulmonary disease, bronchiectasis, asthma, and pulmonary hypertension.

The study was approved by the Clinical Research Ethics Committee of Kartal Dr. Lütfi Kırdar City Hospital on 12 April 2023 (decision no. 2023/514/247/8) and was conducted in accordance with the Declaration of Helsinki. Written informed consent was obtained from all participants.

### 2.3. Measurements and Calculations

Body weight and height were measured using a standard weight and height scale. Body mass index (BMI) was calculated as weight in kilograms divided by height in meters squared (kg/m^2^). BMI was classified according to World Health Organization (WHO) criteria as underweight (<18.5 kg/m^2^), normal weight (18.5–24.9 kg/m^2^), overweight (25.0–29.9 kg/m^2^), and obese (≥30.0 kg/m^2^) [16].

### 2.4. Nutritional and Inflammatory Indices

The Prognostic Nutritional Index (PNI) was calculated using serum albumin and total lymphocyte count according to the following formula: PNI = 10 × serum albumin (g/dL) + 0.005 × total lymphocyte count (/mm^3^) [14].

The Controlling Nutritional Status (CONUT) score was calculated using serum albumin, total lymphocyte count, and total cholesterol levels. The total score ranges from 0 to 12, with higher scores indicating poorer nutritional status. CONUT scores were interpreted as follows: 0–1, normal nutritional status; 2–4, mild malnutrition; 5–8, moderate malnutrition; and 9–12, severe malnutrition [17].

The Nutritional Risk Index (NRI) was calculated using serum albumin and the ratio of current body weight to ideal body weight according to the following formula: NRI = (1.519 × serum albumin, g/dL) + [41.7 × current body weight/ideal body weight]. Lower NRI values indicate a greater risk of malnutrition [18].

Inflammatory indices derived from complete blood count parameters were also calculated. The neutrophil-to-lymphocyte ratio (NLR) was obtained by dividing neutrophil count by lymphocyte count; the monocyte-to-lymphocyte ratio (MLR) by dividing monocyte count by lymphocyte count; the platelet-to-lymphocyte ratio (PLR) by dividing platelet count by lymphocyte count; and the systemic immune-inflammation index (SII) by multiplying platelet count and neutrophil count and dividing the product by lymphocyte count.

### 2.5. Biochemical Analysis

Blood samples for complete blood count (CBC) analysis were collected into dipotassium ethylenediaminetetraacetic acid (EDTA) tubes, whereas samples for biochemical and hormonal analyses were collected into serum tubes without anticoagulant. CBC parameters were analyzed using an automatic hematology analyzer (Sysmex XN-9000 system, Sysmex America, Mundelein, IL, USA). Routine biochemical parameters, including fasting glucose, total cholesterol, high-density lipoprotein (HDL), low-density lipoprotein (LDL), triglycerides (TG), alanine transaminase (ALT), aspartate transaminase (AST), ferritin, total protein, albumin, and C-reactive protein (CRP), were measured using an automated analyzer (COBAS c 702, Roche Diagnostics, Penzberg, Germany). Thyroid-related hormones and antibodies, together with fasting insulin and 25(OH)D, were measured using an automated immunoassay analyzer (COBAS e 801, Roche Diagnostics, Penzberg, Germany).

Insulin resistance was assessed using the homeostasis model assessment of insulin resistance (HOMA-IR), calculated as fasting insulin (μU/mL) × fasting plasma glucose (mg/dL)/405. A HOMA-IR value of ≥2.5 was accepted as indicating insulin resistance [19]. Vitamin D deficiency was defined as a serum 25(OH)D concentration <20 ng/mL, insufficiency as 20–29.99 ng/mL, and sufficiency as ≥30 ng/mL [20].

To better characterize functional heterogeneity within the clinically diagnosed HT population, participants were additionally classified according to biochemical thyroid functional pattern at the time of assessment, based on concurrent TSH, FT4, and FT3 values. Euthyroid pattern was defined as normal TSH, FT4, and FT3; subclinical hypothyroid pattern as elevated TSH with normal FT4 and FT3; overt hypothyroid pattern as elevated TSH with low FT4 and low FT3; subclinical hyperthyroid pattern as low TSH with normal FT4 and FT3; and overt hyperthyroid pattern as low TSH with elevated FT4 and elevated FT3. Cases not meeting these prespecified combinations were categorized as other biochemical patterns. Because information on thyroid medication was limited to yes/no status and dose data were unavailable, this classification was intended to reflect biochemical thyroid status at assessment rather than untreated disease stage.

### 2.6. Statistical Analysis

Statistical analyses were performed using Python (version 3.12.7) packaged by Anaconda, Inc. (Austin, TX, USA) [21]. Data processing was conducted using Pandas (version 2.3.3) [22] and NumPy (version 1.26.4) [23]. The normality of continuous variables was assessed using the Shapiro–Wilk test [24]. Continuous variables are presented as mean ± standard deviation for normally distributed data and as median (interquartile range) for non-normally distributed data. Categorical variables are expressed as number and percentage. Because most variables were not normally distributed, non-parametric tests were primarily used. Comparisons between two independent groups were performed using the Mann–Whitney U test [25], whereas comparisons across more than two groups were conducted using the Kruskal–Wallis test [26]. For variables that met parametric assumptions, the independent-samples *t*-test or one-way analysis of variance (ANOVA) was applied [27]. When an overall non-parametric group difference was significant, post hoc pairwise comparisons were performed using Dunn’s procedure with Bonferroni correction [28]. The chi-square test or Fisher’s exact test was used for categorical variables, as appropriate.

Correlations between nutritional indices, inflammatory markers, and thyroid-related parameters were evaluated using Spearman’s rank correlation coefficient [29]. As a sensitivity analysis, Spearman correlation analyses were repeated after excluding participants with CRP > 10 mg/L, a threshold used to identify individuals with marked systemic inflammation or possible acute inflammatory conditions. This analysis was performed to evaluate whether the observed correlation structure was driven by participants with pronounced inflammatory activity.

Multiple linear regression analyses were used to explore associations between thyroid-related parameters and immunonutritional or inflammatory indices. Because heteroscedasticity was detected in some models and several biochemical variables showed skewed distributions, regression analyses were estimated using heteroscedasticity-consistent robust standard errors (HC3). As a CRP-based sensitivity analysis, the robust regression models were repeated after excluding participants with CRP > 10 mg/L. Additional covariate-adjusted robust regression models were fitted as a robustness analysis by testing each immunonutritional or inflammatory index separately and adjusting for age, BMI, HOMA-IR, vitamin D status, sex, and disease duration. Each index was tested in a separate model to reduce multicollinearity among related indices, particularly among complete blood count-derived inflammatory markers.

Data visualization was performed using Seaborn (version 0.13.2) [30] and Matplotlib (version 3.10.8) [31]. Box plots were used to present the distribution of selected variables across groups, and a heatmap was generated to illustrate the strength and direction of correlations among nutritional and inflammatory indices.

All statistical tests were two-sided, and a *p* value < 0.05 was considered statistically significant. Correlation and regression analyses were conducted using SciPy (version 1.16.3) [32] and Statsmodels (version 0.14.2) [33].

## 3. Results

### 3.1. Descriptive Statistics

Table 1 presents the baseline continuous characteristics of the study population (*n* = 229). The mean age was 42.88 ± 11.58 years, and the mean BMI was 27.68 ± 4.47 kg/m^2^. Thyroid-related parameters showed variability, particularly TSH, which ranged from 0.01 to 96.60 mIU/L. Median serum 25(OH)D was 14.00 ng/mL (IQR: 9.30–21.30). Immunonutritional indices (PNI, NRI, and CONUT) and inflammatory markers (SII, NLR, MLR, and PLR) are also presented in Table 1.

#### 3.1.1. Demographic and Anthropometric Characteristics

The study consisted predominantly of middle-aged adults. Mean age was 42.88 ± 11.58 years, with a median of 43 years (IQR: 16; range: 18–65 years). Mean height and body weight were 161.07 ± 6.36 cm and 71.78 ± 12.09 kg, respectively. The mean BMI was 27.68 ± 4.47 kg/m^2^, and the median BMI was 27.34 kg/m^2^ (IQR: 4.75), indicating that excess body weight was common in the study population.

#### 3.1.2. Metabolic Parameters

Metabolic indicators also showed a broad distribution. Mean fasting insulin was 10.61 ± 5.67 µIU/mL, and mean fasting glucose was 90.23 ± 9.81 mg/dL (median: 90 mg/dL). The mean HOMA-IR value was 2.39 ± 1.38, with a median of 2.10 (IQR: 1.24). Triglycerides were notably variable, with a mean of 141.46 ± 91.02 mg/dL, an IQR of 89.00, and values reaching 1079 mg/dL. Mean total cholesterol, LDL cholesterol, and HDL cholesterol were 195.92 ± 38.40 mg/dL, 118.55 ± 33.54 mg/dL, and 52.62 ± 11.55 mg/dL, respectively.

#### 3.1.3. Liver Enzymes and Protein Status

Liver enzymes were generally within expected ranges. Mean AST and ALT levels were 18.59 ± 6.36 U/L and 17.22 ± 10.26 U/L, respectively, and the median AST/ALT ratio was 1.17 (IQR: 0.43). Mean total protein and albumin values were 71.72 ± 4.09 g/L and 46.01 ± 3.40 g/L, respectively. Ferritin showed marked dispersion, with a mean of 43.22 ± 71.00 ng/mL, a median of 24.60 ng/mL, and a maximum value of 730 ng/mL.

#### 3.1.4. Thyroid Function and Autoimmune Markers

Thyroid-related variables also showed substantial variability. Median TSH was 2.86 mIU/L (IQR: 3.91), whereas the mean TSH was 5.09 ± 9.25 mIU/L, reflecting the right-skewed distribution of this marker. Mean FT3 and FT4 levels were 2.75 ± 0.50 pg/mL and 1.21 ± 0.35 ng/dL, respectively. Mean anti-TPO and anti-TG concentrations were 157.81 ± 191.07 IU/mL and 222.94 ± 559.61 IU/mL, indicating broad inter-individual variation in autoimmune marker levels.

#### 3.1.5. Vitamin D and Inflammatory Markers

Vitamin D levels were generally low. Mean serum vitamin D concentration was 15.63 ± 8.29 ng/mL, with a median of 14.00 ng/mL. Median CRP was 1.72 mg/L (IQR: 3.47), while the mean CRP value was 3.27 ± 4.32 mg/L.

Hematological parameters, including hemoglobin (13.25 ± 6.80 g/dL), white blood cell count (7.77 ± 7.51 ×10^9^/L), platelet count (273.71 ± 68.55 ×10^9^/L), and leukocyte subgroups, were also documented as part of the inflammatory profile.

#### 3.1.6. Derived Nutritional and Inflammatory Indices

The mean PNI score was 46.02 ± 3.40, with a median of 46.01 (IQR: 3.01). The mean NRI score was 119.29 ± 9.31, and the median was 118.74 (IQR: 10.81). CONUT scores were generally low, with a median of 0.00. Mean inflammatory index values were 476.37 ± 245.69 for SII, 1.73 ± 0.69 for NLR, 0.27 ± 0.37 for MLR, and 119.36 ± 36.59 for PLR. Taken together, these descriptive findings suggest a study characterized by excess body weight, low vitamin D status, and considerable variability in metabolic, thyroid-related, autoimmune, and inflammatory markers.

Assessment of distributional assumptions showed that most continuous variables were non-normally distributed (Shapiro–Wilk *p* < 0.05), supporting the use of non-parametric methods for the main analyses. Age (*p* = 0.004); anthropometric variables such as height, weight, and BMI (all *p* < 0.001); and metabolic measures including insulin, glucose, and HOMA-IR (all *p* < 0.001) all deviated from normality. Lipid parameters, liver enzymes, and inflammatory markers showed a similar pattern. Triglyceride values ranged from 35 to 1079 mg/dL and SII values from 76.31 to 1943.24 (both *p* < 0.001), underscoring the skewed distribution of several biomarkers. Among hematological measures, HCT was the only variable consistent with normality (*p* = 0.174), whereas hemoglobin, white blood cell count, platelet count, and leukocyte subgroups all deviated significantly (all *p* < 0.01). Thyroid-related markers were likewise non-normally distributed; TSH ranged from 0.01 to 96.60 mIU/L (*p* < 0.001), FT3 was borderline but still non-normal (*p* = 0.051), and anti-TG values extended up to 4000 IU/mL. PNI, NRI, and CONUT also did not meet normality assumptions, with CONUT scores clustering at lower values.

#### 3.1.7. Categorical Characteristics

Categorical findings are summarized in Table 2. Disease duration was relatively evenly distributed, although the largest subgroup comprised participants with 3–5 years since diagnosis (33.2%). This was followed by 0–2 years (24.5%), 6–8 years (21.8%), and ≥9 years (20.5%).

Excess body weight was common. Half of the study was overweight (50.2%, *n* = 115), 24.5% (*n* = 56) had obesity, and 25.3% (*n* = 58) were within the normal BMI range. Most participants had normal fasting glucose values (87.3%), whereas 12.2% had impaired fasting glucose and only one participant (0.4%) met the ADA criterion for diabetes. Based on HOMA IR classification, 30.1% (*n* = 69) had insulin resistance.

Serum insulin levels were within the reference range for most participants (95.6%), with only small proportions classified as high (3.9%) or low (0.4%). Thyroid function tests also showed that most participants were within the reference range for FT3 (94.3%) and FT4 (83.0%), while TSH was normal in 64.2%, elevated in 27.1%, and low in 8.7%.

Thyroid autoantibody positivity was highly prevalent: anti-TG was elevated in 99.1% of participants and anti-TPO in 80.8%. Vitamin D status was predominantly low, with 70.3% classified as deficient and 20.5% as insufficient; only 9.2% had normal 25(OH)D levels. According to the nutritional indices, all participants had normal NRI scores, whereas 58.1% were classified as having moderate malnutrition by PNI and 10.0% as having mild malnutrition according to CONUT.

When additionally categorized according to biochemical thyroid functional pattern at assessment, most participants were classified as euthyroid (*n* = 132, 57.6%). Smaller proportions were classified as subclinical hypothyroid (*n* = 44, 19.2%), overt hypothyroid (*n* = 3, 1.3%), and subclinical hyperthyroid (*n* = 5, 2.2%), whereas 45 participants (19.7%) showed discordant/other biochemical patterns.

### 3.2. Inferential Statistics

Because most continuous variables were not normally distributed, group comparisons and association analyses were performed primarily with non-parametric tests, mainly the Mann–Whitney U and Kruskal–Wallis tests. Parametric procedures were reserved for variables that met the required assumptions.

Effect sizes were reported alongside *p* values to facilitate interpretation. For Mann–Whitney analyses, effect size was expressed as r (Z/√N), whereas epsilon-squared (ε^2^) was used for Kruskal–Wallis tests. Bonferroni-adjusted post hoc comparisons were applied when the overall group difference was statistically significant.

Table 3 presents the statistically significant between-group findings according to HOMA-IR status, BMI category, and disease duration. The complete set of comparisons, including non-significant results, is provided in Supplementary Table S1.

#### 3.2.1. Results for Nutritional Indices (PNI and NRI)

PNI scores were similar across HOMA-IR class, vitamin D status, and BMI category (all *p* > 0.12), with negligible effect sizes. Disease duration was the only grouping variable associated with PNI (*p* = 0.009), although the effect size was small (ε^2^ = 0.038). Post hoc analysis indicated that participants with a shorter disease duration (0–2 years) had higher PNI values than those in the 6–8 years group (*p* = 0.007).

NRI differed significantly according to HOMA-IR class, with higher values in participants with insulin resistance than in those without (123.50 ± 10.14 vs. 117.47 ± 8.32; *p* < 0.001). The corresponding effect size was moderate (r = −0.313). BMI category was also strongly associated with NRI (*p* < 0.001, ε^2^ = 0.527), and post hoc analyses showed a stepwise increase from normal weight to overweight and obesity. By contrast, NRI did not differ significantly across vitamin D categories (*p* = 0.547, ε^2^ = 0.000) or disease duration groups (*p* = 0.483, ε^2^ = 0.000).

#### 3.2.2. Results for Inflammatory Indices (NLR, MLR, PLR, and SII)

Inflammatory indices were largely stable across the clinical groupings examined. NLR showed no significant differences according to HOMA-IR class (*p* = 0.984, r = −0.001), vitamin D category (*p* = 0.069, ε^2^ = 0.015), BMI category (*p* = 0.776, ε^2^ = 0.000), or disease duration (*p* = 0.661, ε^2^ = 0.000). MLR likewise remained similar across all grouping variables, with ε^2^ values of 0.003 or lower.

PLR and SII also did not vary significantly across HOMA-IR class, vitamin D status, BMI category, or disease duration. For PLR, all *p* values were > 0.50 and effect sizes were negligible. SII values were similarly comparable across HOMA-IR class (*p* = 0.652, r = −0.030), vitamin D category (*p* = 0.229, ε^2^ = 0.004), BMI category (*p* = 0.750, ε^2^ = 0.000), and disease duration (*p* = 0.668, ε^2^ = 0.000).

#### 3.2.3. Results for Thyroid Markers (FT3, FT4, and TSH)

FT3 levels differed significantly according to HOMA-IR class, with slightly higher values in participants with insulin resistance than in those without (2.86 ± 0.51 vs. 2.70 ± 0.49; *p* = 0.027). The effect size was small to moderate (Cohen’s d = −0.321).

Disease duration was also associated with FT3 (*p* = 0.019), although the effect size remained small (η^2^ = 0.043). FT3 did not differ significantly according to vitamin D status or BMI category.

FT4 levels did not vary according to HOMA-IR class (*p* = 0.304, r = 0.068), vitamin D status (*p* = 0.806), or BMI category (*p* = 0.189). Disease duration was associated with FT4 (*p* = 0.033), but again the effect size was small (ε^2^ = 0.025).

TSH levels did not differ significantly according to HOMA-IR class, vitamin D status, BMI category, or disease duration (all *p* > 0.15), and effect sizes were negligible (ε^2^ ≤ 0.010).

#### 3.2.4. Results for Autoimmune Markers (Anti-TPO and Anti-TG)

Neither anti-TPO nor anti-TG differed significantly according to HOMA-IR class, vitamin D category, BMI category, or disease duration. All comparisons were non-significant (*p* > 0.55), and effect sizes were negligible (ε^2^ = 0.000; r ranging from −0.039 to 0.021), despite the wide variability in antibody concentrations.

#### 3.2.5. Results for HOMA-IR Groups

As expected, HOMA-IR values differed markedly according to HOMA-IR classification (*p* < 0.001), with a very large effect size (r = −0.793). Mean HOMA was substantially higher in the insulin resistance group than in the non–insulin resistance group (4.00 ± 1.39 vs. 1.70 ± 0.53).

Vitamin D category was also associated with HOMA-IR (*p* = 0.007), although the effect size was small (ε^2^ = 0.036). Post hoc analysis showed a significant difference between the vitamin D deficiency and insufficiency groups (*p* = 0.010). BMI category was significantly associated with HOMA-IR (*p* < 0.001), with a moderate effect size (ε^2^ = 0.093); both the overweight and obese groups had higher HOMA-IR values than the normal-weight group. No significant difference in HOMA-IR was observed across disease duration groups (*p* = 0.606).

Overall, the statistically significant between-group findings were concentrated in NRI, FT3, FT4, PNI, and HOMA-IR. Participants with insulin resistance had higher NRI and HOMA-IR values and modestly higher FT3 levels. NRI also increased progressively across BMI categories, while disease duration was associated with PNI, FT3, and FT4. In contrast, most inflammatory indices and thyroid autoantibodies did not differ across the examined subgroups.

Box-and-whisker plots illustrating the distribution of indices and biomarkers across vitamin D categories, BMI groups, and disease duration are provided in the Supplementary Material (Supplementary Figures S1–S3).

Participants excluded from the CRP-based sensitivity analyses differed from retained participants primarily in inflammatory markers, with higher CRP, SII, and NLR values, whereas thyroid-related, metabolic, and immunonutritional parameters were generally comparable between groups (Supplementary Table S4).

### 3.3. Correlation Analysis

Spearman’s rank correlation analysis showed strong internal coherence among the inflammatory indices. The strongest positive correlations were observed between SII and NLR (ρ = 0.807) and between SII and PLR (ρ = 0.709). Among the immunonutritional measures, NRI showed a moderate positive correlation with HOMA-IR (ρ = 0.447) and a strong positive correlation with BMI (ρ = 0.768), supporting the interpretation that NRI reflected metabolic and adiposity-related burden more than classical undernutrition risk in this cohort. The full-sample Spearman correlation matrix is presented in Supplementary Figure S4.

For thyroid-related parameters, TSH was inversely correlated with FT4 (ρ = −0.550), and anti-TPO was moderately positively correlated with anti-TG (ρ = 0.522). The correlation between PLR and TSH was weak and negative (ρ = −0.179), consistent with the direction observed in the robust regression models. In contrast, associations between thyroid markers and immunonutritional indices were statistically significant but generally weak, suggesting only partial overlap between nutritional status, inflammatory burden, and thyroid-related abnormalities in adults with HT (Supplementary Figure S4).

As a sensitivity analysis, Spearman correlation analyses were repeated after excluding participants with CRP > 10 mg/L. The overall correlation structure remained stable, with 32 of 35 significant correlations overlapping between the full-sample and CRP-excluded analyses. Strong internal correlations among inflammatory indices were preserved, including SII–NLR (ρ = 0.806) and SII–PLR (ρ = 0.726). The expected inverse TSH–FT4 correlation also remained stable (ρ = −0.559), as did the anti-TPO–anti-TG correlation (ρ = 0.526). Similarly, the strong NRI–BMI correlation (ρ = 0.764) and the moderate NRI–HOMA-IR correlation (ρ = 0.438) were maintained. The weak inverse PLR–TSH correlation also remained in the same direction after CRP-based exclusion (ρ = −0.163). Only a small number of weak correlations changed significance status after excluding participants with marked CRP elevation, and these changes did not materially alter the main correlation pattern. Overall, the CRP-based sensitivity analysis indicated that the principal correlation structure was not primarily driven by participants with CRP > 10 mg/L. The CRP-excluded Spearman correlation matrix is presented in Supplementary Figure S5.

### 3.4. Robust Regression Analysis

Multiple linear regression analyses were conducted to explore the associations between thyroid-related parameters and routinely available immunonutritional and inflammatory indices. Because heteroscedasticity was detected in some preliminary models and several biochemical variables showed skewed distributions, the exploratory regression models were estimated using heteroscedasticity-consistent robust standard errors (HC3). The models were therefore interpreted as exploratory and hypothesis-generating rather than as confirmatory prediction models.

In the robust stepwise regression models, the overall explanatory power remained modest, with R^2^ values ranging from 0.066 to 0.171. In the TSH model, PLR and FT4 were retained as associated variables. PLR showed a weak inverse association with TSH (β = −0.040, HC3 SE = 0.018, *p* = 0.027), while FT4 was also inversely associated with TSH (β = −9.518, HC3 SE = 4.141, *p* = 0.022). This model explained 16.4% of the variance in TSH.

For FT3, TSH and CONUT were retained in the robust exploratory model. TSH was inversely associated with FT3 (β = −0.011, HC3 SE = 0.003, *p* < 0.001), whereas CONUT showed a weak positive association with FT3 (β = 0.097, HC3 SE = 0.041, *p* = 0.019). However, the explanatory power of the FT3 model was low (R^2^ = 0.066), indicating that these associations accounted for only a small proportion of FT3 variability.

In the FT4 model, TSH and FT3 were retained. TSH was inversely associated with FT4 (β = −0.013, HC3 SE = 0.004, *p* = 0.003), while FT3 was positively associated with FT4 (β = 0.129, HC3 SE = 0.056, *p* = 0.021). This model explained 17.1% of the variance in FT4.

For thyroid autoantibodies, Anti-TG and Anti-TPO were reciprocally associated. Anti-TPO was positively associated with Anti-TG (β = 1.104, HC3 SE = 0.305, *p* < 0.001), and Anti-TG was positively associated with Anti-TPO (β = 0.129, HC3 SE = 0.031, *p* < 0.001). Both antibody models explained 14.2% of the variance in the respective dependent variable.

As a sensitivity analysis, the robust stepwise regression models were repeated after excluding participants with CRP > 10 mg/L. Thirteen participants were excluded, leaving 216 participants in the sensitivity analysis. The TSH model remained stable, retaining PLR and FT4 with similar coefficient directions and explanatory power. The FT4 model and antibody-related models were also broadly preserved. By contrast, the FT3 model showed partial sensitivity to CRP-based exclusion: CONUT was no longer retained, while PLR entered the model together with TSH. Therefore, the CONUT–FT3 association observed in the full-sample robust model was interpreted cautiously. The full results of the CRP-based sensitivity regression analysis are presented in Supplementary Table S2.

Additional covariate-adjusted robust regression models were fitted to evaluate whether the index-related associations persisted after adjustment for age, BMI, HOMA-IR, vitamin D status, sex, and disease duration. In these models, each immunonutritional or inflammatory index was tested separately to reduce multicollinearity among related indices. PLR remained associated with TSH both in the full sample and after CRP-based exclusion. Specifically, PLR was inversely associated with TSH in the full sample (β = −0.043, HC3 SE = 0.017, *p* = 0.010) and in the CRP-excluded sample (β = −0.043, HC3 SE = 0.017, *p* = 0.014). The statistically significant findings from the covariate-adjusted robust regression models are provided in Supplementary Table S3. These findings suggest that the PLR–TSH association was the most internally consistent index-related finding. Nevertheless, the modest R^2^ values and the exploratory nature of the models indicate that these indices should be interpreted as supportive markers of within-cohort biochemical heterogeneity rather than as stand-alone predictors of thyroid-related outcomes (Table 4).

## 4. Discussion

In this cross-sectional study conducted in adults with clinically diagnosed HT, we explored within-cohort associations between immunonutritional indices (PNI, NRI, and CONUT), hemogram-derived inflammatory markers (SII, NLR, and PLR), and thyroid function tests and autoimmunity markers, including TSH, FT3, FT4, anti-TPO, and anti-TG. The biological basis of HT lies in an autoimmune response directed against thyroid antigens, while accompanying systemic interactions suggest that the disease may extend beyond thyroid-specific involvement alone. Clinically, HT also exhibits marked phenotypic heterogeneity under the same diagnostic label [2,12,34]. In this context, the present findings suggest that metabolic, immunonutritional, and inflammatory indicators may provide additional information when interpreted alongside conventional thyroid-related markers.

The present study points to a substantial metabolic burden in adults with HT. Meta-analytic evidence linking obesity with thyroid autoimmunity and hypothyroidism suggests that this metabolic profile should be regarded not merely as an accompanying feature, but as a meaningful component of the HT phenotype [35]. In this context, the higher NRI values observed in individuals with insulin resistance (123.50 ± 10.14 vs. 117.47 ± 8.32; *p* < 0.001), together with the progressive increase across BMI categories (*p* < 0.001), indicate that metabolic heterogeneity is clearly reflected in immunonutritional indices. Given that NRI can be calculated without additional cost, it may be considered a practical tool for making the metabolic context more visible in patients with HT.

Vitamin D deficiency was also among the notable findings; the median 25(OH)D level was 14.00 ng/mL, and deficiency was common. Systematic reviews and meta-analyses have shown that low 25(OH)D levels are more frequently observed in HT and have consistently discussed the potential role of vitamin D in immune regulation [36,37]. In addition, meta-analyses of randomized studies suggest that vitamin D supplementation may reduce anti-TPO and anti-TG levels [38,39,40]. Although detailed treatment and supplementation data were not available in the present study, the high prevalence of deficiency suggests that vitamin D status represents an important accompanying factor to be considered in clinical follow-up.

From an immunonutritional perspective, most participants were classified within the normal CONUT category. CONUT is generally regarded as a screening-oriented index based on albumin, cholesterol, and lymphocyte components [13]. Nevertheless, the significant difference observed in PNI according to disease duration (lower in the 6–8-year group compared with the 0–2-year group; *p* = 0.009) suggests that the immunonutritional profile of HT may vary over time. Since PNI combines albumin and lymphocyte count and has been used in a range of clinical risk contexts, this finding may be of interest [14]. In this respect, duration-related variability in PNI suggests that HT does not represent a fully uniform biochemical profile and that some systemic indicators may change over the course of the disease. Similarly, the differences observed in FT3 and FT4 across disease-duration categories (*p* = 0.019 and *p* = 0.033, respectively) further support temporal heterogeneity in thyroid-related parameters.

One of the most internally consistent findings was the association between PLR and TSH in the robust regression analyses. In exploratory regression models estimated using HC3 heteroscedasticity-consistent robust standard errors, the index-related findings were conservative. In the full-sample robust model, PLR and FT4 were retained in the TSH model, with PLR showing a weak inverse association with TSH. This association remained directionally consistent after excluding participants with CRP > 10 mg/L and was also preserved in the covariate-adjusted robust models after adjustment for age, BMI, HOMA-IR, vitamin D status, sex, and disease duration. These findings suggest that the PLR–TSH association was the most internally consistent index-related signal in the present analysis. Studies reporting associations between HT and hemogram-derived inflammatory indices, such as NLR and PLR, also support this interpretation [5,8,41,42]. Nevertheless, the magnitude of this association was weak, and PLR should therefore be interpreted as a supportive marker of within-cohort biochemical heterogeneity rather than as a stand-alone indicator of thyroid status.

The robustness analyses provided a more conservative interpretation of the data. The explanatory power of the robust regression models remained modest, with R^2^ values ranging from 0.066 to 0.171, which is consistent with the multifactorial biological nature of HT. Since the pathogenesis of HT is shaped by the interplay of genetic predisposition, environmental triggers, and immune regulatory processes, a high level of explanatory power from any single index would not be expected [2,12,34]. The Spearman correlation sensitivity analysis also showed that the overall correlation structure remained stable after CRP-based exclusion, with 32 of 35 significant correlations overlapping between the full-sample and CRP-excluded analyses. By contrast, the CONUT–FT3 association observed in the full-sample robust regression model was not retained in the CRP-excluded model, suggesting that it should be interpreted as a less stable, hypothesis-generating finding. Overall, these findings reinforce the supportive rather than stand-alone clinical role of routine inflammatory and immunonutritional indices in HT.

In clinical practice, restricting the evaluation to TSH and FT4 alone may lead to underrecognition of the systemic dimension of HT [12,34]. Current guidelines recommend that clinical findings, biochemical parameters, and autoantibody data should be considered together in the assessment of HT [2]. Anti-TPO and anti-TG positivity are among the principal indicators of the underlying autoimmune activity across the disease spectrum [2,12]. The moderate anti-TG/anti-TPO association observed in this study suggests that these autoantibodies may act in concert within a shared biological network in HT [12]. This, in turn, supports taking autoimmune burden into account during follow-up and interpreting thyroid function together with systemic indicators [12,34]. Thyroid autoantibodies were not interpreted in isolation in the present study; rather, they were evaluated together with thyroid function, metabolic features, vitamin D status, disease duration, and supportive systemic indices in order to better reflect the heterogeneity of clinically diagnosed HT.

HT has a broad clinical spectrum ranging from euthyroidism to overt hypothyroidism [2,12]. This clinical variability may help explain why inflammatory ratios and immunonutritional indices appear more relevant in some subgroups than in others [41,42]. For this reason, indices such as NLR, PLR, and SII may be more useful as complementary tools than as stand-alone diagnostic markers [8,41]. The autoantibody profile, on the other hand, reflects autoimmune burden independently of thyroid function and remains important during follow-up [2,12]. Accordingly, evaluating autoantibody levels together with inflammatory ratios and immunonutritional indices may contribute to a broader characterization of phenotypic diversity in HT [12,41]. However, these findings should be interpreted cautiously until confirmed in prospective studies.

The available evidence indicates that diet-related inflammatory burden and broader dietary patterns are associated with autoimmune thyroid disease and further suggests that diet quality may modulate the HT phenotype [43,44]. Population-based data using immunonutritional indices in relation to thyroid-related outcomes also support the potential relevance of nutritional reserve in endocrine autoimmunity [45]. Meta-analytic evidence further suggests that alterations in the gut microbiota may be present in autoimmune thyroid disease, pointing to an additional systemic axis that may intersect with nutritional and inflammatory profiles [46]. Recent observational data linking obesity with subclinical hypothyroidism and autoimmune thyroid markers are likewise consistent with the metabolic heterogeneity observed in the present study [47]. Overall, these findings support the view that nutritional and inflammatory factors may contribute to the broader clinical profile of HT, although their exact clinical implications remain to be clarified.

## 5. Limitations

This study has several limitations. The cross-sectional design limits causal inference, and the single-center sample may reduce the generalizability of the findings. An additional limitation is the absence of a healthy control group, which restricts disease-specific interpretation of the observed immunonutritional and inflammatory indices. Because information on levothyroxine use was limited to yes/no status and dose data were not available, we were unable to determine whether biochemical thyroid status reflected untreated disease stage, treatment effects, or both. Therefore, the findings should be interpreted as exploratory observations within a clinically diagnosed HT population rather than as disease-specific diagnostic signals. Accordingly, the biochemical functional classification should be interpreted as reflecting thyroid status at assessment rather than untreated disease stage. The absence of disease-control groups with other autoimmune conditions is another important limitation, as it prevents assessment of whether the observed immunonutritional and inflammatory profiles are specific to Hashimoto’s thyroiditis or reflect a broader autoimmune or systemic inflammatory phenotype. Although basic treatment status was available as a yes/no variable, detailed treatment information, including medication type, dose, duration, adherence, dose adjustment, vitamin D supplementation, and dietary intervention, was not available; therefore, treatment-related changes in inflammatory or immunonutritional indices could not be evaluated. Patient-reported symptoms and quality-of-life measures were also not collected, which prevented assessment of whether variation in these indices corresponded to symptom burden, fatigue, mood-related complaints, or other patient-centered outcomes. Future controlled longitudinal studies should include healthy controls and disease-control groups and should evaluate whether treatment or nutritional interventions modify these indices and whether such changes correspond to improvements in patient-reported symptoms.

## 6. Conclusions

In conclusion, this cross-sectional study shows that adults with HT frequently present with vitamin D deficiency, excess body weight, and insulin resistance. Immunonutritional and inflammatory indices showed limited but potentially informative within-cohort associations with thyroid-related and metabolic parameters. NRI appeared to reflect metabolic and adiposity-related burden more than classical undernutrition risk, whereas PLR showed the most internally consistent index-related association with TSH across robust and sensitivity analyses. However, because of the cross-sectional design and the absence of healthy or disease-control groups, these findings should be interpreted as exploratory and hypothesis-generating rather than disease-specific or prognostic. Controlled longitudinal studies are needed to determine whether these indices have clinical relevance for monitoring systemic inflammation, nutritional status, treatment response, or patient-centered outcomes in HT.

## Figures and Tables

**Table 1:** Descriptive Statistics of the Study Population.

Variable	Mean ± SD	Min.–Max.	Median [25–75%]	IQR
Age (years)	42.88 ± 11.58	18.00–65.00	43.00 [35.00–51.00]	16.00
BMI (kg/m^2^)	27.68 ± 4.47	18.67–44.08	27.34 [24.97–29.72]	4.75
Insulin (µIU/mL)	10.61 ± 5.67	1.50–30.20	9.90 [7.00–12.00]	5.00
HOMA-IR	2.39 ± 1.38	0.27–8.37	2.10 [1.49–2.73]	1.24
Triglyceride (mg/dL)	141.46 ± 91.02	35.00–1079.00	122.00 [91.00–180.00]	89.00
TSH (mIU/L)	5.09 ± 9.25	0.01–96.60	2.86 [1.34–5.25]	3.91
FT3 (pg/mL)	2.75 ± 0.50	0.98–4.44	2.73 [2.44–3.04]	0.60
FT4 (ng/dL)	1.21 ± 0.35	0.00–4.01	1.18 [1.03–1.36]	0.33
Anti-TG	222.94 ± 559.61	0.00–4000.00	50.00 [16.60–200.00]	183.40
Anti-TPO	157.81 ± 191.07	2.10–600.00	71.70 [10.00–221.00]	211.00
Vitamin D (ng/mL)	15.63 ± 8.29	3.00–49.60	14.00 [9.30–21.30]	12.00
CRP (mg/L)	3.27 ± 4.32	0.09–33.29	1.72 [0.83–4.30]	3.47
PNI score	46.02 ± 3.40	40.01–81.01	46.01 [44.01–47.02]	3.01
NRI score	119.29 ± 9.31	100.37–172.32	118.74 [113.27–124.08]	10.81
CONUT score	0.53 ± 0.70	0.00–3.00	0.00 [0.00–1.00]	1.00
SII	476.37 ± 245.69	76.31–1943.24	414.72 [319.73–559.98]	240.25
NLR	1.73 ± 0.69	0.42–5.81	1.62 [1.26–1.98]	0.72
MLR	0.27 ± 0.37	0.00–4.78	0.22 [0.19–0.27]	0.09
PLR	119.36 ± 36.59	29.69–250.44	115.98 [94.35–139.74]	45.40

Abbreviations: SD, standard deviation; Min., minimum; Max., maximum; IQR, interquartile range; BMI, body mass index; HOMA-IR, homeostatic model assessment of insulin resistance; TSH, thyroid-stimulating hormone; FT3, free triiodothyronine; FT4, free thyroxine; Anti-TG, anti-thyroglobulin; Anti-TPO, anti-thyroid peroxidase; CRP, C-reactive protein; PNI, Prognostic Nutritional Index; NRI, Nutritional Risk Index; CONUT, Controlling Nutritional Status; SII, systemic immune-inflammation index; NLR, neutrophil-to-lymphocyte ratio; MLR, monocyte-to-lymphocyte ratio; PLR, platelet-to-lymphocyte ratio.

**Table 2:** Categorical Characteristics and Classifications.

Variable	Category	*n*	(%)
Diagnosis Duration	3–5 years	76	33.2
	0–2 years	56	24.5
	6–8 years	50	21.8
	9+ years	47	20.5
BMI class	Overweight	115	50.2
	Normal	58	25.3
	Obesity	56	24.5
HOMA-IR class	No insulin resistance	160	69.9
	Insulin resistance	69	30.1
TSH class	Normal	147	64.2
	High	62	27.1
	Low	20	8.7
Vitamin D class	Deficiency	161	70.3
	Insufficiency	47	20.5
	Normal	21	9.2
PNI score class	Moderate malnutrition	133	58.1
	Normal	54	23.6
	Moderate–severe malnutrition	42	18.3
CONUT score class	Normal	206	90.0
	Mild malnutrition	23	10.0
Thyroid functional status at assessment	Subclinical hypothyroid pattern	44	19.2
	Overt hypothyroid pattern	3	1.3
	Euthyroid pattern	132	57.6
	Subclinical hyperthyroid pattern	5	2.2
	Overt hyperthyroid pattern	0	0.0
	Other biochemical patterns	45	19.7

Abbreviations: BMI, body mass index; HOMA-IR, homeostatic model assessment of insulin resistance; TSH, thyroid-stimulating hormone; PNI, Prognostic Nutritional Index; CONUT, Controlling Nutritional Status. Functional thyroid status at assessment was classified biochemically using concurrent TSH, FT4, and FT3 values. “Other biochemical patterns” refers to discordant biochemical patterns not meeting the prespecified categories.

**Table 3:** Group Comparisons of Nutritional and Inflammatory Indices.

Grouping Variable	Outcome Variable	Result (Mean ± SD)	*p*-Value	Effect Size
HOMA-IR Class	NRI Score (No IR vs. IR)	117.47 ± 8.32 vs. 123.50 ± 10.14	<0.001	r = −0.313
	FT3 (No IR vs. IR)	2.70 ± 0.49 vs. 2.86 ± 0.51	0.027	d = −0.321
BMI Class	NRI Score (Normal)	111.48 ± 5.39	<0.001	ε^2^ = 0.527
	NRI Score (Overweight)	118.49 ± 7.11		
	NRI Score (Obese)	129.03 ± 7.88		
Disease Duration	PNI (0–2 vs. 6–8 years)	46.65 ± 2.32 vs. 45.81 ± 5.68	0.009	ε^2^ = 0.038
	FT3 (0–2 years group)	2.92 ± 0.55	0.019	η^2^ = 0.043
	FT4 (9+ years group)	1.32 ± 0.35	0.033	ε^2^ = 0.025

Continuous variables were compared using the independent-samples *t*-test or Mann–Whitney U test for two-group comparisons and one-way ANOVA or Kruskal–Wallis test for comparisons involving three or more groups, as appropriate. Effect sizes are reported as Cohen’s d (two-group mean comparisons), r (Mann–Whitney U), and epsilon-squared (ε^2^) for Kruskal–Wallis tests. A *p* value < 0.05 was considered statistically significant. Abbreviations: BMI, body mass index; HOMA-IR, homeostatic model assessment of insulin resistance; IR, insulin resistance; NRI, Nutritional Risk Index; PNI, Prognostic Nutritional Index; FT3, free triiodothyronine; FT4, free thyroxine; SD, standard deviation; Effect size metrics are reported as Cohen’s d (d), effect size r, eta-squared (η^2^), or epsilon-squared (ε^2^).

**Table 4:** Exploratory Stepwise Regression Models Using HC3 Robust Standard Errors.

Dependent Variable	Independent Variable	Coefficient (β)	HC3 Robust SE	t	*p*	Model R^2^	Adjusted R^2^
TSH	PLR	−0.040	0.018	−2.21	0.027	0.164	0.156
	FT4	−9.518	4.141	−2.30	0.022		
FT3	TSH	−0.011	0.003	−3.75	<0.001	0.066	0.058
	CONUT	0.097	0.041	2.35	0.019		
FT4	TSH	−0.013	0.004	−3.02	0.003	0.171	0.164
	FT3	0.129	0.056	2.31	0.021		
Anti-TG	Anti-TPO	1.104	0.305	3.62	<0.001	0.142	0.138
Anti-TPO	Anti-TG	0.129	0.031	4.17	<0.001	0.142	0.138

Multiple linear regression models were estimated using HC3 heteroscedasticity-consistent robust standard errors. β indicates the unstandardized regression coefficient; SE, standard error; t, t-statistic; *p*, probability value; R^2^, coefficient of determination. A *p* value < 0.05 was considered statistically significant. Abbreviations: TSH, thyroid-stimulating hormone; FT3, free triiodothyronine; FT4, free thyroxine; Anti-TG, anti-thyroglobulin; Anti-TPO, anti-thyroid peroxidase; PLR, platelet-to-lymphocyte ratio; CONUT, Controlling Nutritional Status; β, unstandardized coefficient; SE, standard error; R^2^, coefficient of determination.

## Data Availability

The data presented in this study are not publicly available due to ethical and privacy restrictions. The data may be made available from the corresponding author upon reasonable request and subject to applicable ethical and institutional approvals.

## Supplementary Materials

The following supporting information can be downloaded at https://www.mdpi.com/article/10.3390/nu18111698/s1. Supplementary File S1: Figure S1: Distributions of indices and biomarkers across vitamin D status categories; Figure S2: Distributions of indices and biomarkers across BMI categories (normal, overweight, obese); Figure S3: Distributions of indices and biomarkers across disease duration categories; Figure S4: Spearman Correlation Heatmap; Figure S5: Spearman correlation heatmap after excluding participants with CRP > 10 mg/L. Supplementary File S2: Table S1: Full subgroup comparisons across all outcomes (median [IQR] and mean ± SD where applicable), including effect sizes and post hoc results.; Table S2: CRP-based sensitivity regression analysis excluding participants with CRP > 10 mg/L; Table S3: Covariate-Adjusted HC3 Robust Regression Models for Index-Related Associations; Table S4: Baseline comparison between participants retained in the CRP-based sensitivity analysis and participants excluded due to CRP > 10 mg/L.
