# Supplementary material for: Immunonutritional Indices, Inflammatory Markers, and Thyroid-Related Parameters in Adults with Hashimoto’s Thyroiditis
Source: Nutrients. 2026 May 26;18(11):1698. doi: 10.3390/nu18111698 (PMC13258694; doi:10.3390/nu18111698)
Supplement: Supplementary file 1 [file nutrients-18-01698-s001.zip › Supplementary File S1.pdf]

Supplementary Figures

S1. Boxplots for Group Comparisons

Patients were categorized into three groups according to serum 25-hydroxyvitamin D levels as vitamin D deficiency, insufficiency, and normal. Comparisons of immunonutritional indices, inflammatory markers, thyroid function parameters, and autoimmune antibody levels across these groups were performed using the Kruskal–Wallis test (Figure S1).

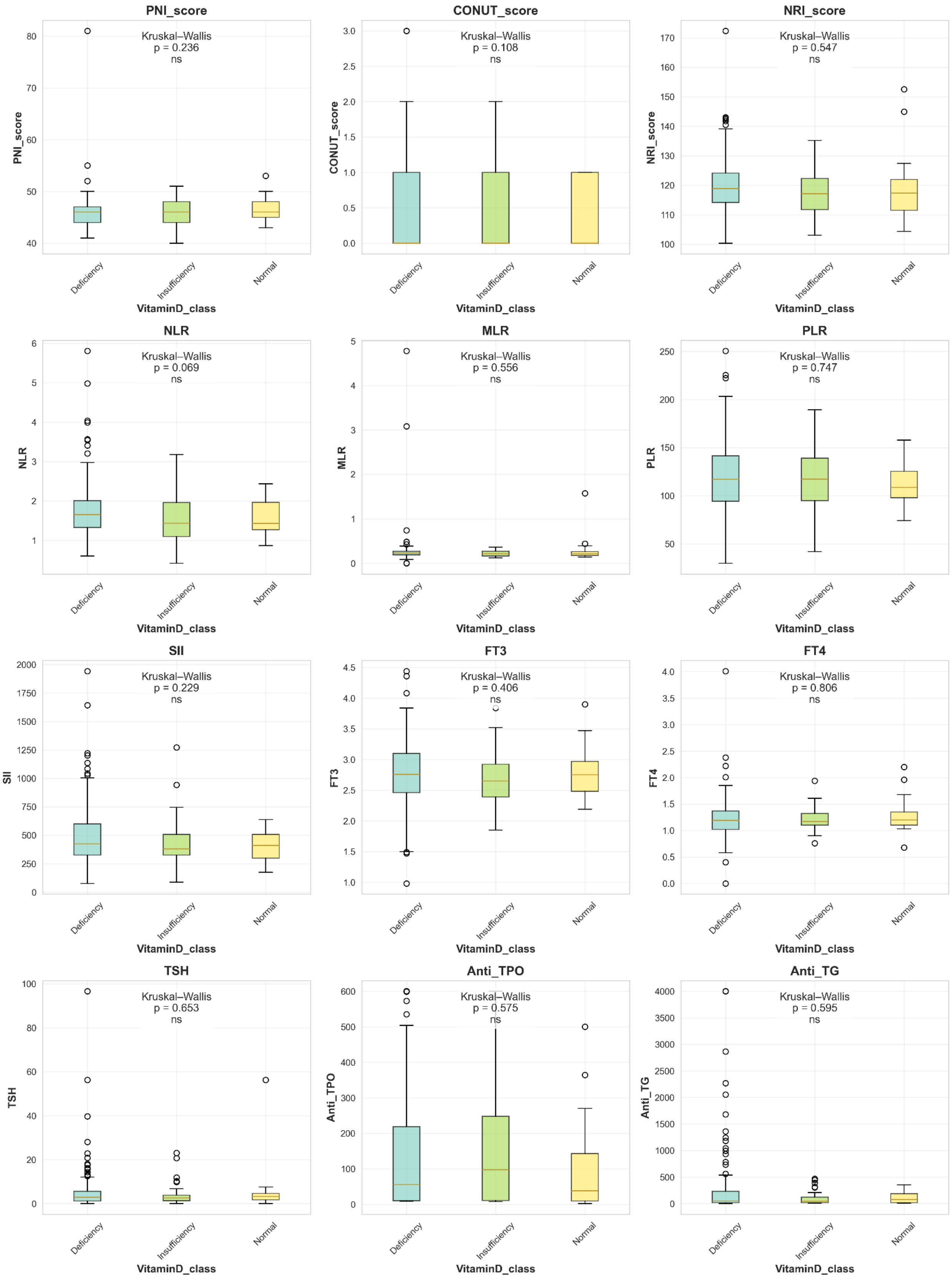

Figure S1. Distributions of indices and biomarkers across vitamin D status categories.

Footnote: Data are visualized using box-and-whisker plots to illustrate the distribution and central tendency of each parameter across different Vitamin D classes. The horizontal line within each box represents the median, while the box boundaries indicate the interquartile range (IQR) from the 25th to the 75th percentile. The whiskers extend to the minimum and maximum values within 1.5 times the IQR; individual circles represent outliers. Group comparisons were performed using the Kruskal–Wallis H test for non-parametric data, with $p < 0.05$ considered statistically significant.

Abbreviations: Anti_TG, antithyroglobulin antibody; Anti_TPO, antithyroid peroxidase antibody; CONUT, controlling nutritional status; FT3, free triiodothyronine; FT4, free thyroxine; MLR, monocyte-to-lymphocyte ratio; NLR, neutrophil-to-lymphocyte ratio; NRI, nutritional risk index; PLR, platelet-to-lymphocyte ratio; PNI, prognostic nutritional index; SII, systemic immune-inflammation index; TSH, thyroid-stimulating hormone.

No statistically significant differences were observed among vitamin D groups in terms of immunonutritional indices. Median PNI, CONUT, and NRI scores were comparable across vitamin D deficiency, insufficiency, and normal groups (PNI: $p = 0.236$; CONUT: $p = 0.108$; NRI: $p = 0.547$), with substantial overlap in interquartile ranges. Similarly, inflammatory indices, including NLR, MLR, PLR, and SII, did not differ significantly among the three vitamin D categories (NLR: $p = 0.069$; MLR: $p = 0.556$; PLR: $p = 0.747$; SII: $p = 0.229$). Although a trend toward higher NLR values was observed in the vitamin D deficiency group, this difference did not reach statistical significance. Assessment of thyroid function tests revealed no significant differences in FT3, FT4, or TSH levels across vitamin D groups (FT3: $p = 0.406$; FT4: $p = 0.806$; TSH: $p = 0.653$).Furthermore, thyroid autoantibody levels demonstrated wide interindividual variability but were not significantly different between vitamin D deficiency, insufficiency, and normal groups. Median anti-TPO and anti-TG levels were similar across all categories (anti-TPO: $p = 0.575$; anti-TG: $p = 0.595$). Overall, stratification by vitamin D status did not reveal statistically significant differences in immunonutritional indices, inflammatory markers, thyroid hormone levels, or thyroid autoantibody titers among patients with Hashimoto's thyroiditis.

Patients were stratified into three groups according to body mass index (BMI) as normal weight, overweight, and obese. Comparisons of immunonutritional indices, inflammatory markers, thyroid function tests, and thyroid autoantibody levels across BMI categories were performed using the Kruskal–Wallis test (Figure S2).

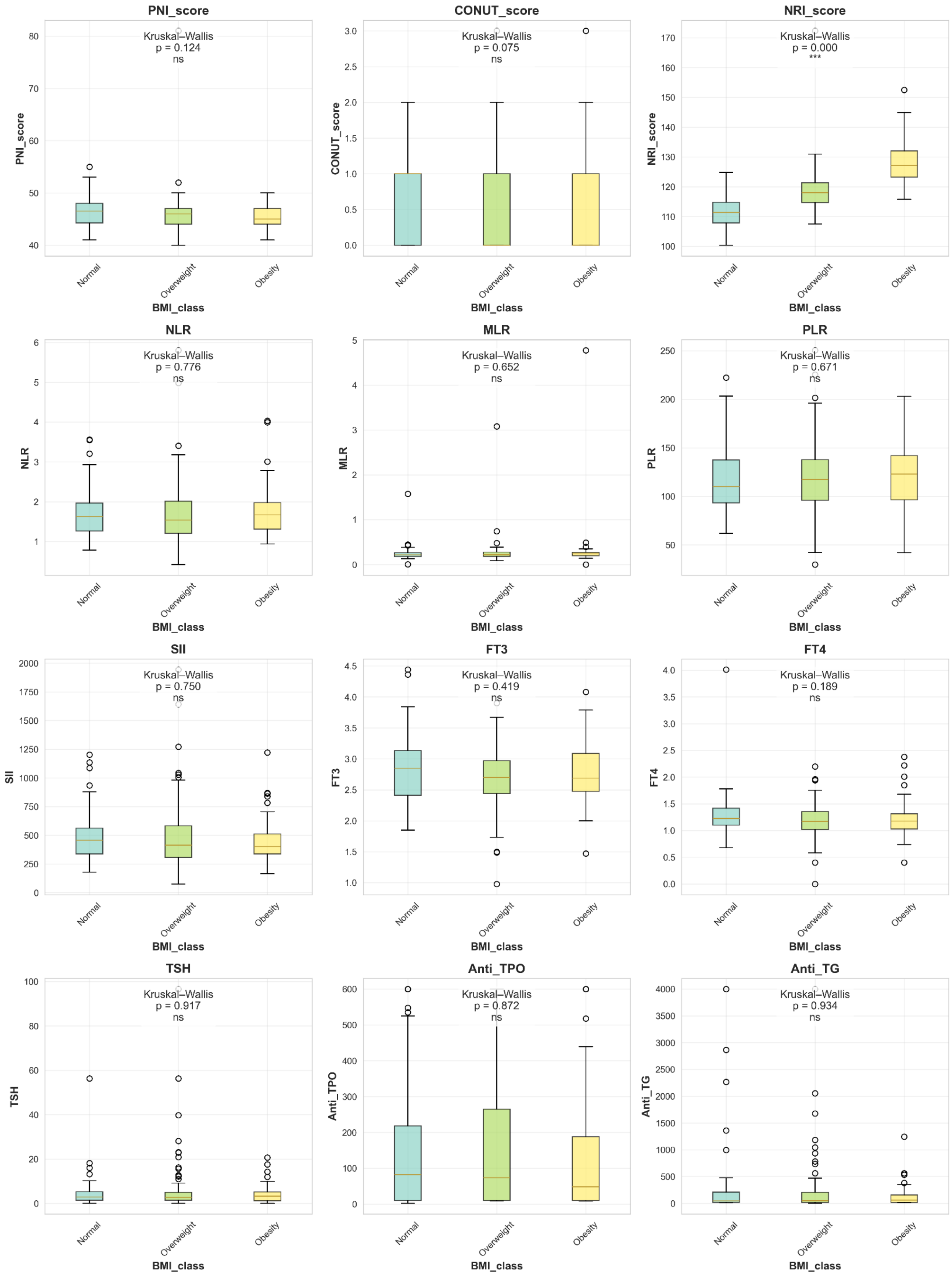

Figure S2. Distributions of indices and biomarkers across BMI categories (normal, overweight, obese).

Footnote: Data are visualized using box-and-whisker plots to illustrate the distribution and central tendency of each parameter across different BMI Classes. The horizontal line within each box represents the median, while the box boundaries indicate the interquartile range (IQR) from the 25th to the 75th percentile. The whiskers extend to the minimum and maximum values within 1.5 times the IQR; individual circles represent outliers. Group comparisons were performed using the Kruskal–Wallis H test for non-parametric data, with $p < 0.05$ considered statistically significant.

Abbreviations: BMI, body mass index; Anti_TG, antithyroglobulin antibody; Anti_TPO, antithyroid peroxidase antibody; CONUT, controlling nutritional status; FT3, free triiodothyronine; FT4, free thyroxine; MLR, monocyte-to-lymphocyte ratio; NLR, neutrophil-to-lymphocyte ratio; NRI, nutritional risk index; PLR, platelet-to-lymphocyte ratio; PNI, prognostic nutritional index; SII, systemic immune-inflammation index; TSH, thyroid-stimulating hormone.

Among immunonutritional indices, PNI and CONUT scores did not differ significantly across BMI categories (PNI: $p = 0.124$; CONUT: $p = 0.075$). In contrast, a statistically significant difference was observed in NRI scores among BMI groups ($p < 0.001$). Median NRI values increased progressively from normal weight to overweight and obese patients, indicating a clear separation between BMI categories. Assessment of inflammatory markers revealed no significant differences in NLR, MLR, PLR, or SII across BMI groups (NLR: $p = 0.776$; MLR: $p = 0.652$; PLR: $p = 0.671$; SII: $p = 0.750$), with overlapping interquartile ranges observed for all indices. Evaluation of thyroid function parameters demonstrated no statistically significant differences in FT3, FT4, or TSH levels among normal-weight, overweight, and obese patients (FT3: $p = 0.419$; FT4: $p = 0.189$; TSH: $p = 0.917$). Similarly, thyroid autoantibody levels did not differ significantly across BMI categories. Median anti-TPO and anti-TG antibody levels were comparable between groups, despite considerable interindividual variability (anti-TPO: $p = 0.872$; anti-TG: $p = 0.934$). Overall, BMI stratification revealed a significant association with NRI, whereas other immunonutritional indices, inflammatory markers, thyroid hormone levels, and thyroid autoantibody titers did not show statistically significant differences across BMI categories.

Patients were stratified into four groups according to disease duration as ≤2 years, 3–5 years, 6–8 years, and ≥9 years since diagnosis. Comparisons of immunonutritional indices, inflammatory markers, thyroid function tests, and thyroid autoantibody levels across disease duration groups were performed using the Kruskal–Wallis test (Figure S3).

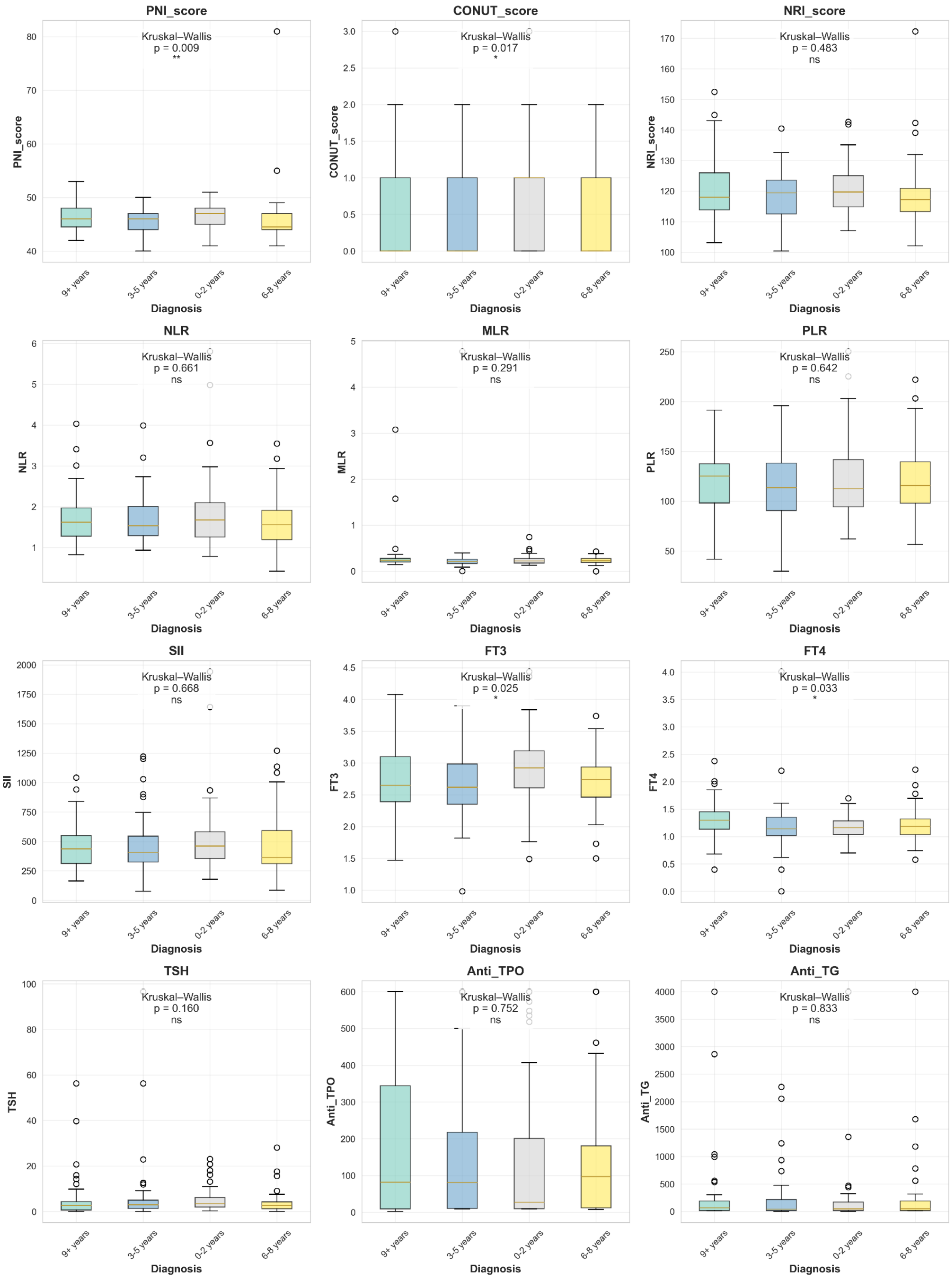

Figure S3. Distributions of indices and biomarkers across disease duration categories.

Footnote: Data are visualized using box-and-whisker plots to illustrate the distribution and central tendency of each parameter across different diagnosis durations. The horizontal line within each box represents the median, while the box boundaries indicate the interquartile range (IQR) from the 25th to the 75th percentile. The whiskers extend to the minimum and maximum values within 1.5 times the IQR; individual circles represent outliers. Group comparisons were performed using the Kruskal–Wallis H test for non-parametric data, with $p < 0.05$ considered statistically significant.

Abbreviations: Anti_TG, antithyroglobulin antibody; Anti_TPO, antithyroid peroxidase antibody; CONUT, controlling nutritional status; FT3, free triiodothyronine; FT4, free thyroxine; MLR, monocyte-to-lymphocyte ratio; NLR, neutrophil-to-lymphocyte ratio; NRI, nutritional risk index; PLR, platelet-to-lymphocyte ratio; PNI, prognostic nutritional index; SII, systemic immune-inflammation index; TSH, thyroid-stimulating hormone.

Among the immunonutritional indices, a statistically significant difference was observed in PNI scores across disease duration groups ($p = 0.009$). Median PNI values varied according to disease duration, with lower values observed particularly in patients with longer disease duration. In addition, CONUT scores also differed significantly among groups ($p = 0.017$). In contrast, NRI scores did not show a statistically significant difference according to disease duration ($p = 0.483$). Evaluation of inflammatory indices revealed no significant differences in NLR, MLR, PLR, or SII across disease duration categories (NLR: $p = 0.661$; MLR: $p = 0.291$; PLR: $p = 0.642$; SII: $p = 0.668$), with overlapping interquartile ranges observed among all groups. Assessment of thyroid hormone levels demonstrated statistically significant differences in both FT3 and FT4 levels according to disease duration (FT3: $p = 0.025$; FT4: $p = 0.033$). Median FT3 and FT4 values varied across duration groups, whereas TSH levels did not differ significantly ($p = 0.160$). Regarding autoimmune parameters, anti-TPO and anti-TG antibody levels showed wide interindividual variability but did not differ significantly across disease duration groups (anti-TPO: $p = 0.752$; anti-TG: $p = 0.833$). Overall, disease duration was associated with significant differences in selected immunonutritional indices (PNI and CONUT) and thyroid hormone levels (FT3 and FT4), whereas inflammatory indices, NRI, TSH, and thyroid autoantibody titers did not demonstrate statistically significant variation across disease duration categories.

S2. Correlation Matrix and Heatmap Visualization

Due to the non-normal distribution of several continuous variables, relationships among thyroid-related, metabolic, immunonutritional, and inflammatory parameters were assessed using Spearman's rank correlation coefficients (ρ). The full-sample Spearman correlation matrix is visualized in Figure S4. As an additional sensitivity analysis, the Spearman correlation analysis was repeated after excluding participants with CRP >10 mg/L to evaluate whether the correlation structure was influenced by individuals with marked systemic inflammation. The CRP-based sensitivity heatmap is presented in Figure S5.

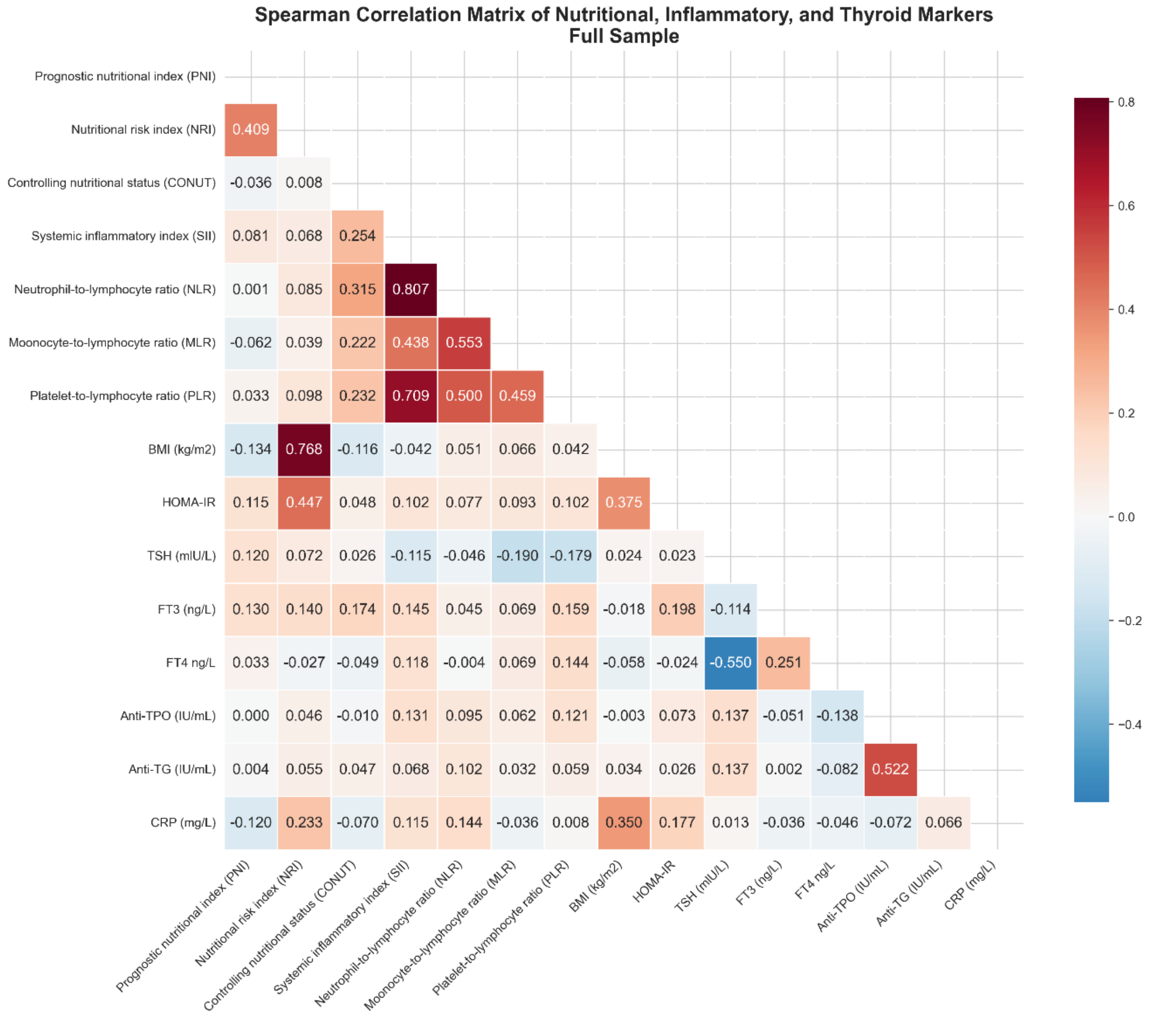

Figure S4. Spearman Correlation Heatmap.

Footnote: The heatmap displays Spearman's rank correlation coefficients (ρ) between nutritional, inflammatory, and thyroid markers in the full sample. The color scale ranges from blue, indicating negative correlations, to dark red, indicating positive correlations, with the coefficient value displayed in each cell.

Abbreviations: Anti-TG, anti-thyroglobulin antibody; Anti-TPO, anti-thyroid peroxidase antibody; BMI, body mass index; CONUT, Controlling Nutritional Status; CRP, C-reactive protein; FT3, free triiodothyronine; FT4, free thyroxine; HOMA-IR, homeostatic model assessment of insulin resistance; MLR, monocyte-to-lymphocyte ratio; NLR, neutrophil-to-lymphocyte ratio; NRI, Nutritional Risk Index; PLR, platelet-to-lymphocyte ratio; PNI, Prognostic Nutritional Index; SII, systemic immune-inflammation index; TSH, thyroid-stimulating hormone.

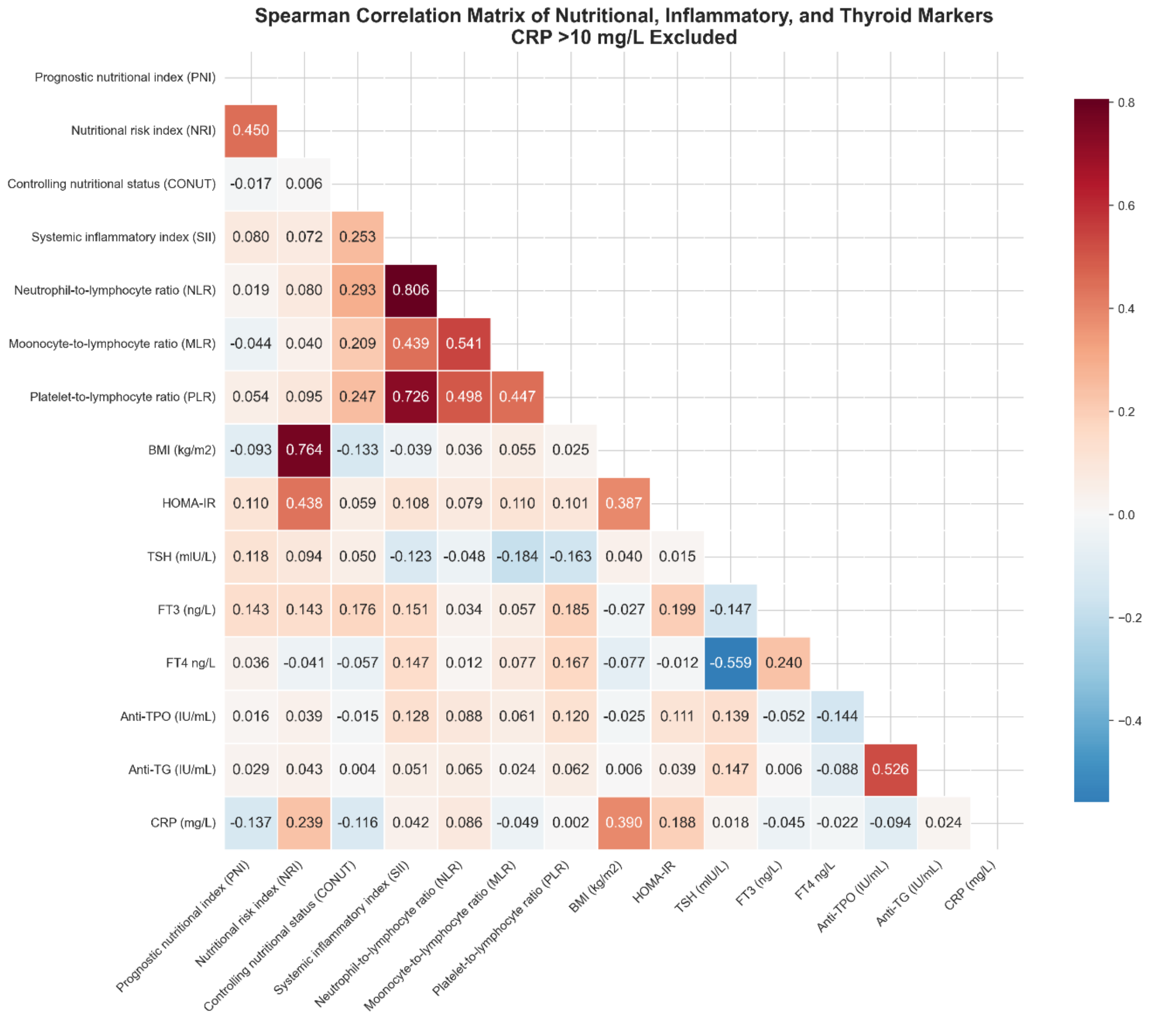

Figure S5. Spearman Correlation Heatmap After Excluding Participants with CRP >10 mg/L.

Footnote: The heatmap displays Spearman's rank correlation coefficients (ρ) after excluding participants with CRP >10 mg/L. This sensitivity analysis was performed to assess whether the main correlation structure was driven by participants with marked CRP elevation. The color scale ranges from blue, indicating negative correlations, to dark red, indicating positive correlations, with the coefficient value displayed in each cell.

Abbreviations: Anti-TG, anti-thyroglobulin antibody; Anti-TPO, anti-thyroid peroxidase antibody; BMI, body mass index; CONUT, Controlling Nutritional Status; CRP, C-reactive protein; FT3, free triiodothyronine; FT4, free thyroxine; HOMA-IR, homeostatic model assessment of insulin resistance; MLR, monocyte-to-lymphocyte ratio; NLR, neutrophil-to-lymphocyte ratio; NRI, Nutritional Risk Index; PLR, platelet-to-lymphocyte ratio; PNI, Prognostic Nutritional Index; SII, systemic immune-inflammation index; TSH, thyroid-stimulating hormone.

Interrelationships Among Inflammatory and Nutritional Indices

Strong and moderate correlations were primarily observed among complete blood count-derived inflammatory indices, indicating substantial internal coherence within this group of markers. SII demonstrated a strong positive correlation with NLR ($\rho = 0.807$, $p < 0.001$) and PLR ($\rho = 0.709$, $p < 0.001$), suggesting that these indices largely capture overlapping inflammatory information. Moderate correlations were also observed between NLR and MLR ($\rho = 0.553$, $p < 0.001$), NLR and PLR ($\rho = 0.500$, $p < 0.001$), and MLR and PLR ($\rho = 0.459$, $p < 0.001$). SII also showed a moderate positive correlation with MLR ($\rho = 0.438$, $p < 0.001$). These findings indicate that

leukocyte- and platelet-derived ratios are closely interrelated and should be interpreted cautiously when used concurrently in multivariable models due to potential redundancy.

The CRP-based sensitivity analysis showed that these interrelationships remained highly stable after excluding participants with CRP >10 mg/L. The SII–NLR correlation was virtually unchanged ($\rho = 0.806$), and the SII–PLR correlation remained strong ($\rho = 0.726$). Similarly, the correlations between NLR and MLR ($\rho = 0.541$), NLR and PLR ($\rho = 0.498$), MLR and PLR ($\rho = 0.447$), and SII and MLR ($\rho = 0.439$) were preserved. These findings suggest that the internal coherence among inflammatory indices was not primarily driven by participants with marked CRP elevation.

Nutritional Status and Metabolic Indicators

Moderate-to-strong associations were identified between nutritional indices and metabolic parameters. NRI showed a strong positive correlation with BMI ($\rho = 0.768$, $p < 0.001$) and a moderate positive correlation with HOMA-IR ($\rho = 0.447$, $p < 0.001$). These findings support the interpretation that, in this cohort, NRI reflected metabolic and adiposity-related burden more strongly than classical undernutrition risk. PNI and NRI were also moderately correlated ($\rho = 0.409$, $p < 0.001$), indicating partial overlap between these nutritional assessment tools. CONUT showed weak-to-moderate correlations with inflammatory markers, suggesting a modest link between nutritional status and systemic inflammatory profiles.

After excluding participants with CRP >10 mg/L, the metabolic and nutritional correlation structure remained largely unchanged. The NRI–BMI correlation remained strong ($\rho = 0.764$), and the NRI–HOMA-IR correlation remained moderate ($\rho = 0.438$). The PNI–NRI correlation also remained moderate and slightly increased in magnitude ($\rho = 0.450$). These sensitivity findings further support the interpretation that the relationship between NRI and metabolic burden was not driven by participants with marked CRP elevation.

Thyroid and Autoimmune Markers

Among thyroid-related parameters, a moderate negative correlation was observed between TSH and FT4 ($\rho = -0.550$, $p < 0.001$), consistent with the expected physiological feedback mechanism of the hypothalamic–pituitary–thyroid axis. FT3 and FT4 were weakly but significantly correlated ($\rho = 0.251$, $p < 0.001$), indicating partial coupling between peripheral thyroid hormones. A moderate positive correlation was found between Anti-TPO and Anti-TG antibodies ($\rho = 0.522$, $p < 0.001$), reflecting shared autoimmune activity in Hashimoto's thyroiditis.

These thyroid-related and autoimmune correlations remained stable after CRP-based exclusion. The inverse TSH–FT4 correlation was preserved ($\rho = -0.559$), as was the Anti-TPO–Anti-TG correlation ($\rho = 0.526$). The FT3–FT4 correlation also remained similar in magnitude ($\rho = 0.240$). These results indicate that the principal thyroid hormone and antibody correlation patterns were not materially influenced by participants with CRP >10 mg/L.

Associations Between Thyroid Parameters and Inflammatory/Nutritional Indices

Several statistically significant but weak correlations were observed between thyroid-related parameters and inflammatory or nutritional indices. FT3 showed weak positive correlations with HOMA-IR ($\rho = 0.198$, $p = 0.003$), PLR ($\rho = 0.159$, $p = 0.016$), and SII ($\rho = 0.145$, $p = 0.028$). TSH

showed weak positive correlations with Anti-TPO ($\rho = 0.137$, $p = 0.038$) and Anti-TG ($\rho = 0.137$, $p = 0.038$), as well as weak negative correlations with MLR ($\rho = -0.190$, $p = 0.004$) and PLR ($\rho = -0.179$, $p = 0.007$). Although statistically significant, the low magnitude of these coefficients suggests limited practical relevance.

The weak inverse PLR–TSH correlation remained in the same direction after excluding participants with CRP >10 mg/L ($\rho = -0.163$). This finding is consistent with the direction observed in the robust regression analyses. The weak positive CONUT–FT3 correlation was also preserved in the CRP-excluded analysis ($\rho = 0.176$), although this association was not retained in the CRP-excluded robust regression model and should therefore be interpreted cautiously. Overall, 32 of 35 significant Spearman correlations overlapped between the full-sample and CRP-excluded analyses. Only a small number of weak correlations changed significance status after CRP-based exclusion, and these changes did not materially alter the main correlation pattern.

Overall, the correlation analysis revealed strong internal associations among inflammatory indices, expected physiological relationships among thyroid hormones, and a stable positive association between thyroid autoantibodies. NRI was strongly associated with BMI and moderately associated with HOMA-IR, supporting its interpretation as a marker closely linked to metabolic and adiposity-related burden in this cohort. In contrast, associations between thyroid parameters and nutritional or inflammatory indices were predominantly weak, indicating that these markers may reflect parallel but only partially overlapping biological processes.

The CRP-based sensitivity analysis further supported the internal consistency of the correlation findings. After excluding participants with CRP >10 mg/L, the strongest inflammatory, metabolic, thyroid-related, and autoimmune correlations were preserved, and the overall correlation structure remained broadly stable. Therefore, the principal correlation pattern was not primarily driven by participants with marked CRP elevation. Nevertheless, weaker correlations involving nutritional and inflammatory indices should be interpreted cautiously and regarded as exploratory.
