# Supplementary material for: Immunonutritional Indices, Inflammatory Markers, and Thyroid-Related Parameters in Adults with Hashimoto’s Thyroiditis
Source: Nutrients. 2026 May 26;18(11):1698. doi: 10.3390/nu18111698 (PMC13258694; doi:10.3390/nu18111698)
Supplement: Supplementary file 1 [file nutrients-18-01698-s001.zip › Supplementary File S2.pdf]

Supplementary Materials

Table S1. Full subgroup comparisons across all outcomes (median [IQR] and mean ± SD where applicable), including effect sizes and post hoc results.

Grouping Variable	Outcome Variable	Group	Mean ± SD	Median [IQR]	n	Test	P value	Effect Size	Effect Size Type	Post-hoc (significant pairs)
HOMA class	PNI score	No insulin resistance	45.83 ± 2.61	46.01 [44.01-47.27]	160	Mann-Whitney U test	0.563	-0.038	r (Mann-Whitney)	
HOMA class	PNI score	Insulin resistance	46.48 ± 4.76	46.01 [44.02-47.02]	69	Mann-Whitney U test	0.563	-0.038	r (Mann-Whitney)	
Vitamin D class	PNI score	Deficiency	45.91 ± 3.71	46.01 [44.01-47.02]	161	Kruskal-Wallis test	0.236	0.004	Epsilon-sq (Kruskal)	
Vitamin D class	PNI score	Insufficiency	46.10 ± 2.62	46.01 [44.01-48.01]	47	Kruskal-Wallis test	0.236	0.004	Epsilon-sq (Kruskal)	
Vitamin D class	PNI score	Normal	46.72 ± 2.35	46.01 [45.01-48.01]	21	Kruskal-Wallis test	0.236	0.004	Epsilon-sq (Kruskal)	
BMI class	PNI score	Normal	46.55 ± 2.82	46.52 [44.27-48.01]	58	Kruskal-Wallis test	0.124	0.010	Epsilon-sq (Kruskal)	
BMI class	PNI score	Overweight	46.00 ± 4.07	46.01 [44.01-47.01]	115	Kruskal-Wallis test	0.124	0.010	Epsilon-sq (Kruskal)	
BMI class	PNI score	Obesity	45.53 ± 2.22	45.02 [44.01-47.01]	56	Kruskal-Wallis test	0.124	0.010	Epsilon-sq (Kruskal)	
Diagnosis	PNI score	9+ years	46.16 ± 2.67	46.01 [44.51-48.01]	47	Kruskal-Wallis test	0.009	0.038	Epsilon-sq (Kruskal)	0-2 years vs 6-8 years (p=0.007); 6-8 years vs 0-2 years (p=0.007)
Diagnosis	PNI score	3-5 years	45.62 ± 2.30	46.01 [44.01-47.01]	76	Kruskal-Wallis test	0.009	0.038	Epsilon-sq (Kruskal)	0-2 years vs 6-8 years (p=0.007);

										6-8 years vs 0-2 years (p=0.007)
Diagnosis	PNI score	0-2 years	46.65 ± 2.32	47.01 [45.01-48.01]	56	Kruskal-Wallis test	0.009	0.038	Epsilon-sq (Kruskal)	0-2 years vs 6-8 years (p=0.007); 6-8 years vs 0-2 years (p=0.007)
Diagnosis	PNI score	6-8 years	45.81 ± 5.68	44.52 [44.01-47.01]	50	Kruskal-Wallis test	0.009	0.038	Epsilon-sq (Kruskal)	0-2 years vs 6-8 years (p=0.007); 6-8 years vs 0-2 years (p=0.007)
HOMA class	NRI score	No insulin resistance	117.47 ± 8.32	116.45 [111.68-121.45]	160	Mann-Whitney U test	0.000	-0.313	r (Mann-Whitney)	
HOMA class	NRI score	Insulin resistance	123.50 ± 10.14	121.69 [117.45-127.70]	69	Mann-Whitney U test	0.000	-0.313	r (Mann-Whitney)	
Vitamin D class	NRI score	Deficiency	119.71 ± 9.41	118.91 [114.15-124.17]	161	Kruskal-Wallis test	0.547	0.000	Epsilon-sq (Kruskal)	
Vitamin D class	NRI score	Insufficiency	117.84 ± 7.60	117.11 [111.72-122.33]	47	Kruskal-Wallis test	0.547	0.000	Epsilon-sq (Kruskal)	
Vitamin D class	NRI score	Normal	119.35 ± 11.82	117.39 [111.51-121.98]	21	Kruskal-Wallis test	0.547	0.000	Epsilon-sq (Kruskal)	
BMI class	NRI score	Normal	111.48 ± 5.39	111.39 [107.87-114.77]	58	Kruskal-Wallis test	0.000	0.527	Epsilon-sq (Kruskal)	Normal vs Obesity (p=0.000); Normal vs Overweight (p=0.000); Obesity vs Normal (p=0.000); Obesity vs Overweight (p=0.000); Overweight vs Normal (p=0.000);

										Overweight vs Obesity (p=0.000)
BMI class	NRI score	Overweight	118.49 ± 7.11	118.04 [114.72-121.36]	115	Kruskal-Wallis test	0.000	0.527	Epsilon-sq (Kruskal)	Normal vs Obesity (p=0.000); Normal vs Overweight (p=0.000); Obesity vs Normal (p=0.000); Obesity vs Overweight (p=0.000); Overweight vs Normal (p=0.000); Overweight vs Obesity (p=0.000)
BMI class	NRI score	Obesity	129.03 ± 7.88	127.18 [123.22-132.02]	56	Kruskal-Wallis test	0.000	0.527	Epsilon-sq (Kruskal)	Normal vs Obesity (p=0.000); Normal vs Overweight (p=0.000); Obesity vs Normal (p=0.000); Obesity vs Overweight (p=0.000); Overweight vs Normal (p=0.000); Overweight vs Obesity (p=0.000)
Diagnosis	NRI score	9+ years	120.65 ± 10.74	118.01 [113.87-125.97]	47	Kruskal-Wallis test	0.483	0.000	Epsilon-sq (Kruskal)	
Diagnosis	NRI score	3-5 years	117.99 ± 8.10	119.41 [112.53-123.59]	76	Kruskal-Wallis test	0.483	0.000	Epsilon-sq (Kruskal)	
Diagnosis	NRI score	0-2 years	120.26 ± 7.99	119.71 [114.87-125.09]	56	Kruskal-Wallis test	0.483	0.000	Epsilon-sq (Kruskal)	

Diagnosis	NRI score	6-8 years	118.90 ± 10.83	117.22 [113.27-120.94]	50	Kruskal-Wallis test	0.483	0.000	Epsilon-sq (Kruskal)	
HOMA class	NLR	No insulin resistance	1.73 ± 0.69	1.62 [1.26-2.01]	160	Mann-Whitney U test	0.984	-0.001	r (Mann-Whitney)	
HOMA class	NLR	Insulin resistance	1.72 ± 0.70	1.59 [1.29-1.97]	69	Mann-Whitney U test	0.984	-0.001	r (Mann-Whitney)	
Vitamin D class	NLR	Deficiency	1.79 ± 0.73	1.65 [1.33-2.01]	161	Kruskal-Wallis test	0.069	0.015	Epsilon-sq (Kruskal)	
Vitamin D class	NLR	Insufficiency	1.57 ± 0.62	1.44 [1.10-1.96]	47	Kruskal-Wallis test	0.069	0.015	Epsilon-sq (Kruskal)	
Vitamin D class	NLR	Normal	1.56 ± 0.45	1.44 [1.28-1.97]	21	Kruskal-Wallis test	0.069	0.015	Epsilon-sq (Kruskal)	
BMI class	NLR	Normal	1.71 ± 0.63	1.63 [1.26-1.97]	58	Kruskal-Wallis test	0.776	0.000	Epsilon-sq (Kruskal)	
BMI class	NLR	Overweight	1.72 ± 0.76	1.54 [1.20-2.01]	115	Kruskal-Wallis test	0.776	0.000	Epsilon-sq (Kruskal)	
BMI class	NLR	Obesity	1.75 ± 0.63	1.67 [1.31-1.98]	56	Kruskal-Wallis test	0.776	0.000	Epsilon-sq (Kruskal)	
Diagnosis	NLR	9+ years	1.73 ± 0.64	1.62 [1.28-1.97]	47	Kruskal-Wallis test	0.661	0.000	Epsilon-sq (Kruskal)	
Diagnosis	NLR	3-5 years	1.69 ± 0.54	1.53 [1.29-2.01]	76	Kruskal-Wallis test	0.661	0.000	Epsilon-sq (Kruskal)	
Diagnosis	NLR	0-2 years	1.86 ± 0.92	1.67 [1.26-2.10]	56	Kruskal-Wallis test	0.661	0.000	Epsilon-sq (Kruskal)	
Diagnosis	NLR	6-8 years	1.64 ± 0.67	1.56 [1.19-1.91]	50	Kruskal-Wallis test	0.661	0.000	Epsilon-sq (Kruskal)	
HOMA class	MLR	No insulin resistance	0.27 ± 0.38	0.22 [0.18-0.27]	160	Mann-Whitney U test	0.487	-0.046	r (Mann-Whitney)	

HOMA class	MLR	Insulin resistance	0.28 ± 0.35	0.23 [0.19-0.27]	69	Mann-Whitney U test	0.487	-0.046	r (Mann-Whitney)	
Vitamin D class	MLR	Deficiency	0.28 ± 0.43	0.23 [0.19-0.27]	161	Kruskal-Wallis test	0.556	0.000	Epsilon-sq (Kruskal)	
Vitamin D class	MLR	Insufficiency	0.22 ± 0.06	0.22 [0.16-0.27]	47	Kruskal-Wallis test	0.556	0.000	Epsilon-sq (Kruskal)	
Vitamin D class	MLR	Normal	0.29 ± 0.30	0.22 [0.18-0.26]	21	Kruskal-Wallis test	0.556	0.000	Epsilon-sq (Kruskal)	
BMI class	MLR	Normal	0.25 ± 0.19	0.22 [0.18-0.26]	58	Kruskal-Wallis test	0.652	0.000	Epsilon-sq (Kruskal)	
BMI class	MLR	Overweight	0.26 ± 0.28	0.22 [0.19-0.28]	115	Kruskal-Wallis test	0.652	0.000	Epsilon-sq (Kruskal)	
BMI class	MLR	Obesity	0.32 ± 0.61	0.24 [0.20-0.27]	56	Kruskal-Wallis test	0.652	0.000	Epsilon-sq (Kruskal)	
Diagnosis	MLR	9+ years	0.33 ± 0.46	0.24 [0.20-0.28]	47	Kruskal-Wallis test	0.291	0.003	Epsilon-sq (Kruskal)	
Diagnosis	MLR	3-5 years	0.28 ± 0.53	0.21 [0.17-0.26]	76	Kruskal-Wallis test	0.291	0.003	Epsilon-sq (Kruskal)	
Diagnosis	MLR	0-2 years	0.24 ± 0.10	0.22 [0.18-0.27]	56	Kruskal-Wallis test	0.291	0.003	Epsilon-sq (Kruskal)	
Diagnosis	MLR	6-8 years	0.23 ± 0.07	0.24 [0.19-0.28]	50	Kruskal-Wallis test	0.291	0.003	Epsilon-sq (Kruskal)	
HOMA class	PLR	No insulin resistance	118.40 ± 37.06	113.49 [94.33-138.99]	160	Mann-Whitney U test	0.538	-0.041	r (Mann-Whitney)	
HOMA class	PLR	Insulin resistance	121.59 ± 35.63	119.75 [96.13-139.91]	69	Mann-Whitney U test	0.538	-0.041	r (Mann-Whitney)	
Vitamin D class	PLR	Deficiency	120.81 ± 38.47	116.96 [94.34-141.55]	161	Kruskal-Wallis test	0.747	0.000	Epsilon-sq (Kruskal)	

Vitamin D class	PLR	Insufficiency	117.36 ± 34.73	117.37 [94.81-139.04]	47	Kruskal-Wallis test	0.747	0.000	Epsilon-sq (Kruskal)	
Vitamin D class	PLR	Normal	112.73 ± 23.92	108.56 [97.74-125.26]	21	Kruskal-Wallis test	0.747	0.000	Epsilon-sq (Kruskal)	
BMI class	PLR	Normal	117.52 ± 37.28	110.18 [93.15-137.51]	58	Kruskal-Wallis test	0.671	0.000	Epsilon-sq (Kruskal)	
BMI class	PLR	Overweight	119.94 ± 36.91	117.35 [95.96-137.65]	115	Kruskal-Wallis test	0.671	0.000	Epsilon-sq (Kruskal)	
BMI class	PLR	Obesity	120.08 ± 35.79	122.90 [96.38-141.93]	56	Kruskal-Wallis test	0.671	0.000	Epsilon-sq (Kruskal)	
Diagnosis	PLR	9+ years	122.13 ± 33.27	125.17 [98.20-137.52]	47	Kruskal-Wallis test	0.642	0.000	Epsilon-sq (Kruskal)	
Diagnosis	PLR	3-5 years	114.52 ± 36.15	113.49 [90.72-138.13]	76	Kruskal-Wallis test	0.642	0.000	Epsilon-sq (Kruskal)	
Diagnosis	PLR	0-2 years	121.37 ± 40.21	112.42 [94.33-141.73]	56	Kruskal-Wallis test	0.642	0.000	Epsilon-sq (Kruskal)	
Diagnosis	PLR	6-8 years	121.88 ± 36.33	115.84 [98.09-139.61]	50	Kruskal-Wallis test	0.642	0.000	Epsilon-sq (Kruskal)	
HOMA class	SII	No insulin resistance	471.26 ± 244.80	431.76 [309.34-550.94]	160	Mann-Whitney U test	0.652	-0.030	r (Mann-Whitney)	
HOMA class	SII	Insulin resistance	488.24 ± 249.12	410.50 [347.33-570.82]	69	Mann-Whitney U test	0.652	-0.030	r (Mann-Whitney)	
Vitamin D class	SII	Deficiency	498.52 ± 264.14	424.61 [325.15-599.43]	161	Kruskal-Wallis test	0.229	0.004	Epsilon-sq (Kruskal)	
Vitamin D class	SII	Insufficiency	429.80 ± 208.64	380.61 [325.83-507.29]	47	Kruskal-Wallis test	0.229	0.004	Epsilon-sq (Kruskal)	
Vitamin D class	SII	Normal	410.78 ± 127.65	411.24 [299.63-507.28]	21	Kruskal-Wallis test	0.229	0.004	Epsilon-sq (Kruskal)	

BMI class	SII	Normal	486.52 ± 226.38	456.91 [336.94-561.89]	58	Kruskal-Wallis test	0.750	0.000	Epsilon-sq (Kruskal)	
BMI class	SII	Overweight	483.41 ± 278.20	414.36 [307.75-582.06]	115	Kruskal-Wallis test	0.750	0.000	Epsilon-sq (Kruskal)	
BMI class	SII	Obesity	451.41 ± 189.34	400.76 [337.02-511.37]	56	Kruskal-Wallis test	0.750	0.000	Epsilon-sq (Kruskal)	
Diagnosis	SII	9+ years	464.79 ± 199.52	436.66 [313.88-550.47]	47	Kruskal-Wallis test	0.668	0.000	Epsilon-sq (Kruskal)	
Diagnosis	SII	3-5 years	458.60 ± 211.67	407.26 [325.97-545.20]	76	Kruskal-Wallis test	0.668	0.000	Epsilon-sq (Kruskal)	
Diagnosis	SII	0-2 years	514.25 ± 306.72	461.97 [355.55-582.41]	56	Kruskal-Wallis test	0.668	0.000	Epsilon-sq (Kruskal)	
Diagnosis	SII	6-8 years	471.85 ± 258.92	363.69 [311.53-592.21]	50	Kruskal-Wallis test	0.668	0.000	Epsilon-sq (Kruskal)	
HOMA class	FT3	No insulin resistance	2.70 ± 0.49	2.69 [2.39-3.00]	160	Independent t-test	0.027	-0.321	Cohen's d	
HOMA class	FT3	Insulin resistance	2.86 ± 0.51	2.80 [2.54-3.12]	69	Independent t-test	0.027	-0.321	Cohen's d	
Vitamin D class	FT3	Deficiency	2.76 ± 0.53	2.76 [2.46-3.10]	161	One-way ANOVA	0.535	0.006	Eta-squared	
Vitamin D class	FT3	Insufficiency	2.68 ± 0.41	2.65 [2.39-2.92]	47	One-way ANOVA	0.535	0.006	Eta-squared	
Vitamin D class	FT3	Normal	2.81 ± 0.42	2.75 [2.48-2.97]	21	One-way ANOVA	0.535	0.006	Eta-squared	
BMI class	FT3	Normal	2.84 ± 0.56	2.85 [2.41-3.14]	58	Kruskal-Wallis test	0.419	0.000	Epsilon-sq (Kruskal)	
BMI class	FT3	Overweight	2.69 ± 0.47	2.70 [2.44-2.97]	115	Kruskal-Wallis test	0.419	0.000	Epsilon-sq (Kruskal)	

BMI class	FT3	Obesity	2.78 ± 0.49	2.69 [2.47-3.09]	56	Kruskal-Wallis test	0.419	0.000	Epsilon-sq (Kruskal)	
Diagnosis	FT3	9+ years	2.73 ± 0.47	2.65 [2.39-3.10]	47	One-way ANOVA	0.019	0.043	Eta-squared	
Diagnosis	FT3	3-5 years	2.65 ± 0.48	2.62 [2.35-2.98]	76	One-way ANOVA	0.019	0.043	Eta-squared	
Diagnosis	FT3	0-2 years	2.92 ± 0.55	2.92 [2.61-3.19]	56	One-way ANOVA	0.019	0.043	Eta-squared	
Diagnosis	FT3	6-8 years	2.73 ± 0.45	2.74 [2.46-2.94]	50	One-way ANOVA	0.019	0.043	Eta-squared	
HOMA class	FT4	No insulin resistance	1.22 ± 0.36	1.21 [1.04-1.37]	160	Mann-Whitney U test	0.304	0.068	r (Mann-Whitney)	
HOMA class	FT4	Insulin resistance	1.19 ± 0.32	1.16 [1.02-1.28]	69	Mann-Whitney U test	0.304	0.068	r (Mann-Whitney)	
Vitamin D class	FT4	Deficiency	1.21 ± 0.38	1.19 [1.02-1.37]	161	Kruskal-Wallis test	0.806	0.000	Epsilon-sq (Kruskal)	
Vitamin D class	FT4	Insufficiency	1.20 ± 0.20	1.17 [1.10-1.32]	47	Kruskal-Wallis test	0.806	0.000	Epsilon-sq (Kruskal)	
Vitamin D class	FT4	Normal	1.27 ± 0.33	1.20 [1.10-1.35]	21	Kruskal-Wallis test	0.806	0.000	Epsilon-sq (Kruskal)	
BMI class	FT4	Normal	1.28 ± 0.44	1.23 [1.10-1.42]	58	Kruskal-Wallis test	0.189	0.006	Epsilon-sq (Kruskal)	
BMI class	FT4	Overweight	1.17 ± 0.30	1.17 [1.02-1.35]	115	Kruskal-Wallis test	0.189	0.006	Epsilon-sq (Kruskal)	
BMI class	FT4	Obesity	1.23 ± 0.33	1.17 [1.03-1.31]	56	Kruskal-Wallis test	0.189	0.006	Epsilon-sq (Kruskal)	
Diagnosis	FT4	9+ years	1.32 ± 0.35	1.30 [1.14-1.45]	47	Kruskal-Wallis test	0.033	0.025	Epsilon-sq (Kruskal)	

Diagnosis	FT4	3-5 years	1.19 ± 0.44	1.14 [1.02-1.35]	76	Kruskal-Wallis test	0.033	0.025	Epsilon-sq (Kruskal)	
Diagnosis	FT4	0-2 years	1.17 ± 0.21	1.16 [1.04-1.29]	56	Kruskal-Wallis test	0.033	0.025	Epsilon-sq (Kruskal)	
Diagnosis	FT4	6-8 years	1.20 ± 0.30	1.19 [1.04-1.32]	50	Kruskal-Wallis test	0.033	0.025	Epsilon-sq (Kruskal)	
HOMA class	TSH	No insulin resistance	5.48 ± 10.52	2.56 [1.27-5.27]	160	Mann-Whitney U test	0.531	-0.042	r (Mann-Whitney)	
HOMA class	TSH	Insulin resistance	4.18 ± 5.20	3.15 [1.79-4.89]	69	Mann-Whitney U test	0.531	-0.042	r (Mann-Whitney)	
Vitamin D class	TSH	Deficiency	5.35 ± 9.89	2.92 [1.25-5.59]	161	Kruskal-Wallis test	0.653	0.000	Epsilon-sq (Kruskal)	
Vitamin D class	TSH	Insufficiency	3.86 ± 4.64	2.59 [1.31-3.85]	47	Kruskal-Wallis test	0.653	0.000	Epsilon-sq (Kruskal)	
Vitamin D class	TSH	Normal	5.78 ± 11.76	3.25 [1.74-4.68]	21	Kruskal-Wallis test	0.653	0.000	Epsilon-sq (Kruskal)	
BMI class	TSH	Normal	4.74 ± 7.80	2.87 [1.33-5.27]	58	Kruskal-Wallis test	0.917	0.000	Epsilon-sq (Kruskal)	
BMI class	TSH	Overweight	5.70 ± 11.47	2.68 [1.37-4.94]	115	Kruskal-Wallis test	0.917	0.000	Epsilon-sq (Kruskal)	
BMI class	TSH	Obesity	4.18 ± 4.16	3.17 [1.22-5.14]	56	Kruskal-Wallis test	0.917	0.000	Epsilon-sq (Kruskal)	
Diagnosis	TSH	9+ years	5.50 ± 10.20	2.68 [0.65-4.36]	47	Kruskal-Wallis test	0.160	0.010	Epsilon-sq (Kruskal)	
Diagnosis	TSH	3-5 years	5.69 ± 12.69	2.93 [1.36-5.04]	76	Kruskal-Wallis test	0.160	0.010	Epsilon-sq (Kruskal)	
Diagnosis	TSH	0-2 years	5.02 ± 4.94	3.42 [2.00-6.19]	56	Kruskal-Wallis test	0.160	0.010	Epsilon-sq (Kruskal)	

Diagnosis	TSH	6-8 years	3.85 ± 4.93	2.61 [1.21-4.31]	50	Kruskal-Wallis test	0.160	0.010	Epsilon-sq (Kruskal)	
HOMA class	Anti TPO	No insulin resistance	155.51 ± 191.12	72.20 [9.72-212.50]	160	Mann-Whitney U test	0.553	-0.039	r (Mann-Whitney)	
HOMA class	Anti TPO	Insulin resistance	163.15 ± 192.24	70.30 [11.30-278.00]	69	Mann-Whitney U test	0.553	-0.039	r (Mann-Whitney)	
Vitamin D class	Anti TPO	Deficiency	165.27 ± 200.95	55.70 [10.20-219.00]	161	Kruskal-Wallis test	0.575	0.000	Epsilon-sq (Kruskal)	
Vitamin D class	Anti TPO	Insufficiency	154.08 ± 175.29	97.70 [10.75-248.00]	47	Kruskal-Wallis test	0.575	0.000	Epsilon-sq (Kruskal)	
Vitamin D class	Anti TPO	Normal	108.93 ± 139.50	38.00 [9.68-143.00]	21	Kruskal-Wallis test	0.575	0.000	Epsilon-sq (Kruskal)	
BMI class	Anti TPO	Normal	166.32 ± 199.70	81.90 [10.20-218.00]	58	Kruskal-Wallis test	0.872	0.000	Epsilon-sq (Kruskal)	
BMI class	Anti TPO	Overweight	161.73 ± 193.05	73.70 [9.88-265.00]	115	Kruskal-Wallis test	0.872	0.000	Epsilon-sq (Kruskal)	
BMI class	Anti TPO	Obesity	140.94 ± 179.90	48.60 [9.72-187.75]	56	Kruskal-Wallis test	0.872	0.000	Epsilon-sq (Kruskal)	
Diagnosis	Anti TPO	9+ years	193.21 ± 212.39	81.80 [9.00-344.00]	47	Kruskal-Wallis test	0.752	0.000	Epsilon-sq (Kruskal)	
Diagnosis	Anti TPO	3-5 years	158.49 ± 189.61	81.30 [10.20-217.50]	76	Kruskal-Wallis test	0.752	0.000	Epsilon-sq (Kruskal)	
Diagnosis	Anti TPO	0-2 years	139.17 ± 190.53	27.30 [9.76-200.50]	56	Kruskal-Wallis test	0.752	0.000	Epsilon-sq (Kruskal)	
Diagnosis	Anti TPO	6-8 years	144.36 ± 173.10	96.90 [12.20-180.75]	50	Kruskal-Wallis test	0.752	0.000	Epsilon-sq (Kruskal)	
HOMA class	Anti TG	No insulin resistance	196.52 ± 468.97	49.25 [16.80-196.25]	160	Mann-Whitney U test	0.750	0.021	r (Mann-Whitney)	

HOMA class	Anti TG	Insulin resistance	284.19 ± 728.23	61.50 [14.60-232.00]	69	Mann-Whitney U test	0.750	0.021	r (Mann-Whitney)	
Vitamin D class	Anti TG	Deficiency	275.57 ± 656.78	50.00 [16.70-234.00]	161	Kruskal-Wallis test	0.595	0.000	Epsilon-sq (Kruskal)	
Vitamin D class	Anti TG	Insufficiency	95.78 ± 120.20	42.60 [17.25-124.50]	47	Kruskal-Wallis test	0.595	0.000	Epsilon-sq (Kruskal)	
Vitamin D class	Anti TG	Normal	104.08 ± 104.20	80.00 [15.50-189.00]	21	Kruskal-Wallis test	0.595	0.000	Epsilon-sq (Kruskal)	
BMI class	Anti TG	Normal	355.21 ± 859.28	45.50 [16.50-209.75]	58	Kruskal-Wallis test	0.934	0.000	Epsilon-sq (Kruskal)	
BMI class	Anti TG	Overweight	200.06 ± 472.49	47.10 [16.70-206.50]	115	Kruskal-Wallis test	0.934	0.000	Epsilon-sq (Kruskal)	
BMI class	Anti TG	Obesity	132.94 ± 206.11	61.40 [16.42-155.75]	56	Kruskal-Wallis test	0.934	0.000	Epsilon-sq (Kruskal)	
Diagnosis	Anti TG	9+ years	286.93 ± 716.40	64.80 [17.45-191.50]	47	Kruskal-Wallis test	0.833	0.000	Epsilon-sq (Kruskal)	
Diagnosis	Anti TG	3-5 years	190.30 ± 388.73	41.50 [15.47-217.75]	76	Kruskal-Wallis test	0.833	0.000	Epsilon-sq (Kruskal)	
Diagnosis	Anti TG	0-2 years	190.28 ± 556.82	47.25 [16.90-171.50]	56	Kruskal-Wallis test	0.833	0.000	Epsilon-sq (Kruskal)	
Diagnosis	Anti TG	6-8 years	248.99 ± 623.36	51.95 [16.50-193.50]	50	Kruskal-Wallis test	0.833	0.000	Epsilon-sq (Kruskal)	
HOMA class	HOMA	No insulin resistance	1.70 ± 0.53	1.76 [1.26-2.16]	160	Mann-Whitney U test	0.000	-0.793	r (Mann-Whitney)	
HOMA class	HOMA	Insulin resistance	4.00 ± 1.39	3.66 [2.93-4.72]	69	Mann-Whitney U test	0.000	-0.793	r (Mann-Whitney)	
Vitamin D class	HOMA	Deficiency	2.57 ± 1.47	2.21 [1.69-2.93]	161	Kruskal-Wallis test	0.007	0.036	Epsilon-sq (Kruskal)	Deficiency vs Insufficiency

										(p=0.010); Insufficiency vs Deficiency (p=0.010)
Vitamin D class	HOMA	Insufficiency	1.94 ± 0.98	1.70 [1.18-2.45]	47	Kruskal-Wallis test	0.007	0.036	Epsilon-sq (Kruskal)	Deficiency vs Insufficiency (p=0.010); Insufficiency vs Deficiency (p=0.010)
Vitamin D class	HOMA	Normal	2.07 ± 1.15	1.88 [1.45-2.44]	21	Kruskal-Wallis test	0.007	0.036	Epsilon-sq (Kruskal)	Deficiency vs Insufficiency (p=0.010); Insufficiency vs Deficiency (p=0.010)
BMI class	HOMA	Normal	1.80 ± 0.94	1.58 [1.23-2.15]	58	Kruskal-Wallis test	0.000	0.093	Epsilon-sq (Kruskal)	Normal vs Obesity (p=0.000); Normal vs Overweight (p=0.002); Obesity vs Normal (p=0.000); Overweight vs Normal (p=0.002)
BMI class	HOMA	Overweight	2.44 ± 1.36	2.16 [1.64-2.89]	115	Kruskal-Wallis test	0.000	0.093	Epsilon-sq (Kruskal)	Normal vs Obesity (p=0.000); Normal vs Overweight (p=0.002); Obesity vs Normal (p=0.000); Overweight vs Normal (p=0.002)
BMI class	HOMA	Obesity	2.92 ± 1.56	2.44 [1.90-3.68]	56	Kruskal-Wallis test	0.000	0.093	Epsilon-sq (Kruskal)	Normal vs Obesity (p=0.000); Normal vs Overweight

										(p=0.002); Obesity vs Normal (p=0.000); Overweight vs Normal (p=0.002)
Diagnosis	HOMA	9+ years	2.11 ± 0.93	2.02 [1.50-2.48]	47	Kruskal-Wallis test	0.606	0.000	Epsilon-sq (Kruskal)	
Diagnosis	HOMA	3-5 years	2.47 ± 1.52	2.12 [1.52-2.79]	76	Kruskal-Wallis test	0.606	0.000	Epsilon-sq (Kruskal)	
Diagnosis	HOMA	0-2 years	2.40 ± 1.43	2.07 [1.42-2.72]	56	Kruskal-Wallis test	0.606	0.000	Epsilon-sq (Kruskal)	
Diagnosis	HOMA	6-8 years	2.55 ± 1.44	2.24 [1.65-2.96]	50	Kruskal-Wallis test	0.606	0.000	Epsilon-sq (Kruskal)	

Footnote: Data are presented as Mean ± SD or Median [IQR]. The normality of data distribution was assessed using the Shapiro-Wilk test. For comparisons between two groups, the Independent t-test was used for parametric data and the Mann-Whitney U test for non-parametric data. For comparisons among three or more groups, One-way ANOVA (parametric) or the Kruskal-Wallis H test (non-parametric) was performed. Post-hoc analyses were conducted using the Dunn-Bonferroni test following significant Kruskal-Wallis results. Effect sizes are reported as Cohen's d, r, Eta-squared, or Epsilon-squared where appropriate. Significance was defined as $p < 0.05$. Post-hoc analyses were only performed for variables showing a statistically significant difference in the initial omnibus test (Kruskal-Wallis or ANOVA) involving more than two groups. The "Post-hoc (significant pairs)" column lists only the specific group pairings that demonstrated a statistically significant difference following multiple comparison adjustments; empty cells indicate no significant differences were found between any pairs.
Abbreviations: Anti-TG, antithyroglobulin antibody; Anti-TPO, antithyroid peroxidase antibody; BMI, body mass index; FT3, free triiodothyronine; FT4, free thyroxine; HOMA-IR, homeostatic model assessment of insulin resistance; IQR, interquartile range; MLR, monocyte-to-lymphocyte ratio; NLR, neutrophil-to-lymphocyte ratio; NRI, nutritional risk index; PLR, platelet-to-lymphocyte ratio; PNI, prognostic nutritional index; SD, standard deviation; SII, systemic immune-inflammation index; TSH, thyroid-stimulating hormone.

Table S2. CRP-Based Sensitivity Analysis Excluding Participants with CRP >10 mg/L: Exploratory Stepwise Regression Models Using HC3 Robust Standard Errors.

Dependent Variable	Independent Variable	β	HC3 Robust SE	t	p-value	95% CI Lower	95% CI Upper	Model R^2	Adjusted R^2	N
TSH	PLR	−0.040	0.019	−2.05	0.040	−0.078	−0.002	0.159	0.152	216
TSH	FT4	−9.509	4.411	−2.16	0.031	−18.154	−0.864	0.159	0.152	216
FT3	TSH	−0.010	0.003	−3.64	<0.001	−0.016	−0.005	0.082	0.073	216
FT3	PLR	0.002	0.001	2.46	0.014	0.000	0.004	0.082	0.073	216
FT4	TSH	−0.012	0.004	−3.05	0.002	−0.020	−0.004	0.163	0.156	216
FT4	FT3	0.123	0.062	1.98	0.048	0.001	0.244	0.163	0.156	216
Anti-TG	Anti-TPO	0.960	0.268	3.58	<0.001	0.435	1.486	0.124	0.120	216
Anti-TPO	Anti-TG	0.129	0.040	3.20	0.001	0.050	0.208	0.124	0.120	216

Footnote: Participants with CRP >10 mg/L were excluded from this sensitivity analysis. Thirteen participants were excluded, leaving 216 participants in the analytic sample. Regression models were estimated using HC3 heteroscedasticity-consistent robust standard errors. β indicates the unstandardized regression coefficient. **Abbreviations**: CRP, C-reactive protein; TSH, thyroid-stimulating hormone; FT3, free triiodothyronine; FT4, free thyroxine; Anti-TG, anti-thyroglobulin; Anti-TPO, anti-thyroid peroxidase; PLR, platelet-to-lymphocyte ratio.

Supplementary Table S2 presents the CRP-based sensitivity regression analysis after excluding participants with CRP >10 mg/L. This analysis was performed to evaluate whether the exploratory regression findings were driven by participants with marked systemic inflammation.

Table S3. Covariate-Adjusted HC3 Robust Regression Models for Index-Related Associations.

Analysis	Dependent Variable	Index Tested	β	HC3 Robust SE	t	p-value	95% CI Lower	95% CI Upper	Model R^2	Adjusted R^2	N
Full sample	TSH	PLR	−0.043	0.017	−2.58	0.010	−0.076	−0.010	0.086	0.049	229
Full sample	Anti-TPO	MLR	84.952	43.111	1.97	0.049	0.457	169.446	0.053	0.014	229
CRP >10 excluded	TSH	PLR	−0.043	0.017	−2.46	0.014	−0.077	−0.009	0.092	0.052	216
CRP >10 excluded	FT3	PLR	0.002	0.001	2.45	0.014	0.000	0.003	0.163	0.126	216

Footnote: Only statistically significant index-related associations are shown. Each index was tested in a separate covariate-adjusted model. Models were adjusted for age, BMI, HOMA-IR, vitamin D status, sex, and disease duration and estimated using HC3 heteroscedasticity-consistent robust standard errors. β indicates the unstandardized regression coefficient. **Abbreviations**: TSH, thyroid-stimulating hormone; FT3, free triiodothyronine; Anti-TPO, anti-thyroid peroxidase; PLR, platelet-to-lymphocyte ratio; MLR, monocyte-to-lymphocyte ratio; CRP, C-reactive protein.

Supplementary Table S3 presents statistically significant index-related associations from the covariate-adjusted robust regression models used as an additional robustness analysis. Each immunonutritional or inflammatory index was entered into a separate model to reduce multicollinearity among related indices, particularly among complete blood count-derived inflammatory markers. All models were adjusted for age, BMI, HOMA-IR, vitamin D status, sex, and disease duration. Non-significant models are not displayed in this table.

Table S4. Baseline Comparison Between Participants Retained in the CRP-Based Sensitivity Analysis and Participants Excluded Due to CRP >10 mg/L.

Supplementary Table S4 compares participants retained in the CRP-based sensitivity analyses with those excluded due to CRP >10 mg/L. This comparison was performed to characterize whether the excluded participants differed systematically from the retained analytic sample.

**Variable**	**Retained: CRP ≤10 mg/L (n = 216)**	**Excluded: CRP >10 mg/L (n = 13)**	**Test**	**p-value**
Age	42.00 [34.00–50.00]	51.00 [42.00–58.00]	Mann–Whitney U test	0.084
BMI (kg/m²)	27.29 [24.96–29.73]	28.30 [26.10–31.21]	Mann–Whitney U test	0.237
HOMA-IR	2.10 [1.49–2.73]	2.29 [1.63–3.04]	Mann–Whitney U test	0.333
Vitamin D (µg/L)	14.00 [9.30–21.30]	15.90 [9.80–21.50]	Mann–Whitney U test	0.760
TSH (mIU/L)	2.89 [1.34–5.30]	2.44 [1.34–4.47]	Mann–Whitney U test	0.774
FT3 (ng/L)	2.73 [2.43–3.04]	2.80 [2.50–3.02]	Mann–Whitney U test	0.835
FT4 (ng/L)	1.18 [1.03–1.36]	1.18 [1.07–1.25]	Mann–Whitney U test	0.935
Anti-TPO (IU/mL)	70.95 [10.00–220.25]	89.00 [10.00–245.00]	Mann–Whitney U test	0.525
Anti-TG (IU/mL)	44.60 [16.50–196.25]	189.00 [50.00–241.00]	Mann–Whitney U test	0.084
CRP (mg/L)	1.60 [0.80–3.59]	11.39 [10.77–20.47]	Mann–Whitney U test	<0.001
PNI	46.01 [44.00–47.02]	46.01 [44.01–47.03]	Mann–Whitney U test	0.911
NRI	118.61 [113.07–123.98]	120.28 [116.10–126.70]	Mann–Whitney U test	0.260
CONUT	0.00 [0.00–1.00]	0.00 [0.00–1.00]	Mann–Whitney U test	0.988
SII	407.52 [315.17–547.93]	667.20 [439.74–782.78]	Mann–Whitney U test	0.003
NLR	1.59 [1.23–1.97]	2.09 [1.63–2.84]	Mann–Whitney U test	0.008
PLR	115.38 [94.21–138.49]	126.27 [105.80–149.78]	Mann–Whitney U test	0.286
MLR	0.22 [0.19–0.27]	0.24 [0.21–0.29]	Mann–Whitney U test	0.133

Note: Values are presented as median [interquartile range]. Continuous variables were compared using the Mann–Whitney U test. Participants with CRP >10 mg/L were excluded in the CRP-based sensitivity analyses.
Abbreviations: CRP, C-reactive protein; BMI, body mass index; HOMA-IR, homeostatic model assessment of insulin resistance; TSH, thyroid-stimulating hormone; FT3, free triiodothyronine; FT4, free thyroxine; Anti-TPO, anti-thyroid peroxidase; Anti-TG, anti-thyroglobulin; PNI, Prognostic Nutritional Index; NRI, Nutritional Risk Index; CONUT, Controlling Nutritional Status; SII, systemic immune-inflammation index; NLR, neutrophil-to-lymphocyte ratio; PLR, platelet-to-lymphocyte ratio; MLR, monocyte-to-lymphocyte ratio.
